# *HLA-G* Gene Variability Is Associated with Papillary Thyroid Carcinoma Morbidity and the HLA-G Protein Profile

**DOI:** 10.3390/ijms241612858

**Published:** 2023-08-16

**Authors:** Bruna C. Bertol, Guilherme Debortoli, Fabrício C. Dias, Jéssica N. G. de Araújo, Luana S. M. Maia, Bibiana S. de Almeida, Nathalie L. de Figueiredo-Feitosa, Luiz Carlos C. de Freitas, Erick C. Castelli, Celso T. Mendes-Junior, Vivian N. Silbiger, Léa M. Z. Maciel, Eduardo A. Donadi

**Affiliations:** 1 Postgraduate Program of Basic and Applied Immunology, Ribeirão Preto Medical School, University of São Paulo, Ribeirão Preto 14049-900, Brazil; 2 Princess Margaret Cancer Centre, University Health Network, Toronto, ON M5G 2M9, Canada; 3 Department of Anthropology, University of Toronto at Mississauga, Mississauga, ON L5L 1C6, Canada; guilherme.debortoli@mail.utoronto.ca; 4 Division of Clinical Immunology, Department of Medicine, Ribeirão Preto Medical School, University of São Paulo, Ribeirão Preto 14049-900, Brazil; diasfc@gmail.com (F.C.D.); luana.sellamottamaia@unibas.ch (L.S.M.M.); bibianasgorla@hotmail.com (B.S.d.A.); 5 Department of Clinical Analysis and Toxicology, Federal University of Rio Grande do Norte, Natal 59012-570, Brazil; jessnaysub@hotmail.com (J.N.G.d.A.); viviansilbiger@hotmail.com (V.N.S.); 6 Division of Endocrinology and Metabolism, Department of Medicine, Ribeirão Preto Medical School, University of São Paulo, Ribeirão Preto 14049-900, Brazil; nathaliefigueiredo@yahoo.com.br (N.L.d.F.-F.); leamaciel@hcrp.usp.br (L.M.Z.M.); 7 Department of Ophthalmology, Otorhinolaryngology and Head and Neck Surgery, Ribeirão Preto Medical School, University of São Paulo, Ribeirão Preto 14049-900, Brazil; lcconti@fmrp.usp.br; 8 Department of Pathology, School of Medicine, São Paulo State University, Botucatu 18618-687, Brazil; erick.castelli@unesp.br; 9 Departamento de Química, Faculdade de Filosofia, Ciências e Letras de Ribeirão Preto, Universidade de São Paulo, Ribeirão Preto 14049-900, Brazil; ctmendes@ffclrp.usp.br

**Keywords:** papillary thyroid carcinoma, HLA-G, haplotypes

## Abstract

Human leukocyte antigen (HLA)-G is an immune checkpoint molecule that is highly expressed in papillary thyroid carcinoma (PTC). The *HLA-G* gene presents several functional polymorphisms distributed across the coding and regulatory regions (5′URR: 5′ upstream regulatory region and 3′UTR: 3′ untranslated region) and some of them may impact HLA-G expression and human malignancy. To understand the contribution of the *HLA-G* genetic background in PTC, we studied the *HLA-G* gene variability in PTC patients in association with tumor morbidity, HLA-G tissue expression, and plasma soluble (sHLA-G) levels. We evaluated 185 PTC patients and 154 healthy controls. Polymorphic sites defining coding, regulatory and extended haplotypes were characterized by sequencing analyses. HLA-G tissue expression and plasma soluble HLA-G levels were evaluated by immunohistochemistry and ELISA, respectively. Compared to the controls, the *G*0104a_(5′URR)_G*01:04:04_(coding)_UTR-03_(3’UTR)_ extended haplotype was underrepresented in the PTC patients, while *G*0104a_(5′URR)_G*01:04:01_(coding)_UTR-03_(3′UTR)_ was less frequent in patients with metastatic and multifocal tumors. Decreased HLA-G tissue expression and undetectable plasma sHLA-G were associated with the *G*010102a_(5′URR)_G*01:01:02:01_(coding)_UTR-02_(3′UTR)_ extended haplotype. We concluded that the *HLA-G* variability was associated with PTC development and morbidity, as well as the magnitude of the encoded protein expression at local and systemic levels.

## 1. Introduction

Thyroid cancer (TC) is the most common endocrine malignancy, and its incidence has markedly increased during the past few decades [1]. Differentiated thyroid carcinomas (DTCs) are the most frequent ones, originating from thyroid follicular cells. Among DTCs, papillary thyroid carcinoma (PTC) comprises about 80% of cases [2]. Although the majority of PTC patients present a good prognosis when adequately treated with the standard therapy (i.e., surgery and selective use of radioactive iodine (RAI)) [3], some patients present an aggressive tumor behavior [4]. Due to the frequent inconclusive diagnosis of TC, a substantial number of people undergo unnecessary partial or total thyroidectomy, with them being exposed to an avoidable risk [5].

Over the past two decades, considerable progress has been made in the understanding of the molecular mechanisms associated with DTC development. The elucidation of genetic and epigenetic events affecting key signaling pathways, such as the mitogen-activated protein kinase (MAPK) pathway, and the role of these mediators in thyroid carcinogenesis has brought beneficial clinical management to DTC patients [6]. More recently, the development of kinase inhibition-based therapies, which allow for the targeting of tumor cells, are considered a novel tool for personalized medicine in TC [7]. Despite this progress, there are still limitations concerning the detection of malignant cases and the prediction of unfavorable outcomes [4]. Studies on TC have reported several candidate genes, including those associated with the regulation of inflammation and the immune response [8].

Human leucocyte antigen-G (HLA-G) is a unique non-classical immunomodulatory major histocompatibility complex (MHC) class Ib molecule [9] that is physiologically expressed in the thymus, cornea, pancreas, erythroid and endothelial precursors, and in trophoblastic cells at the maternal–fetal interface. In pathological conditions, HLA-G is expressed in a great number of tumors [10], particularly in PTC [11,12]. The mechanisms that stimulate HLA-G expression in the context of cancer are complex, depending on the individual’s genetic background [13] and various environmental factors such as hypoxia, stress, hormones, and specific cytokines [14]. HLA-G expression in tumor cells is deleterious to the host because of its interaction with leukocyte receptors, particularly ILT-2 and ILT-4, which downregulate the cytotoxic activity of CD8^+^ T and natural killer (NK) cells and inhibit antigen presentation, lymphocyte proliferation, and antibody formation [15,16]. Alternative splicing membrane-bound (HLA-G1, -G2, -G3, and -G4) and soluble (HLA-G5, -G6, and -G7) HLA-G isoforms may also present immunomodulatory properties [17].

The main feature that distinguishes *HLA-G* from classical class I genes is the limited variability at the coding region. The overall structure of the *HLA-G* alleles has been maintained during the evolution process, in which most of its variable sites are synonymous mutations or are observed in introns [18]. Currently, 117 alleles encoding 38 different proteins have been reported by the International Immunogenetics Database (IMGT, 3.52.0) [19,20,21]. Nucleotide/amino acid variability at the coding region may produce conformational changes in the molecule, which may modify its principal functions, i.e., interaction with cell receptors, isoform production, modulation of the immune response, and the ability to couple peptides [13]. On the other hand, nucleotide variability at the *HLA-G* regulatory regions (5′ upstream regulatory region: 5′URR and 3′ untranslated region: 3′UTR) may potentially influence the targeting of transcriptional and post-transcriptional elements, affecting the *HLA-G* gene and protein expression [22,23]. Accordingly, these variation sites along the *HLA-G* gene have been reported in pre-eclampsia [24], cancer [25,26], transplants [27], and autoimmune [28,29], chronic inflammatory [30], and infectious disorders [31,32]. In some of these conditions, the *HLA-G* gene variability potentially affected the magnitude of HLA-G expression [23,33,34].

Considering that (i) HLA-G is a well-recognized immune checkpoint molecule [9], (ii) HLA-G is expressed in several thyroidal disorders, especially in PTC, but it is not in histologically normal thyroid specimens [12], (iii) the *HLA-G* gene has several nucleotide variation sites at the regulatory regions that have been associated with the magnitude of molecule production [18], and (iv) the study of the *HLA-G* variation sites in PTC has mostly focused at the 3′UTR of the gene [35], we evaluated the complete *HLA-G* gene variability (5′URR, coding, 3′UTR, and extended haplotypes) in PTC patients in association with the clinical, histopathological, and tumor features and with the HLA-G protein expression profile. We identified *HLA-G*-specific regions and/or extended haplotypes associated with: (i) PTC histological and prognosis features, (ii) HLA-G expression in tumor specimens, and (iii) soluble plasma HLA-G detection before thyroidectomy.

## 2. Results

### 2.1. HLA-G Gene Variability in PTC Patients and Control Subjects

For *HLA-G* gene nucleotide variability analyses, we evaluated 151 PTC patients and 154 unrelated healthy controls from the same geographical region. All detected *HLA-G* polymorphic sites fit the Hardy–Weinberg expectations and present strong linkage disequilibrium (LD) (*p* < 0.05), indicating that the alleles do not segregate randomly, justifying the haplotype inferences. Association analyses were conducted considering the three major *HLA-G* gene segments, taken individually (5′URR, coding, and 3’UTR) (Table S1), and as extended (5′URR/coding/3′UTR) haplotypes (Table 1).

Considering the *HLA-G* 5′URR, 19 different haplotypes were found, 14 in PTC patients and 17 in the controls. The *G*010102a haplotype was the most frequent (32.95%), followed by *G*010101a (21.15%) in the total population. Compared to the control group, the frequency of the *G*0104a haplotype was underrepresented in PTC patients (odds ratio (OR) = 0.5273, 95% confidence interval (95% CI) = 0.3221–0.8632, *p* = 0.0109).

Twenty-three different *HLA-G* coding region haplotypes were observed for the whole group of individuals, of which 17 were observed among the PTC patients and 20 were observed among the controls. In total, the G*01:01:01:01 haplotype was the most frequent (20.66%), followed by G*01:01:02:01 (17.21%). PTC patients presented a lower frequency of the G*01:04:04 haplotype when compared to the controls (OR = 0.4488, 95% CI = 0.2221–0.9068, *p* = 0.0285).

A total of 14 different *HLA-G* 3′UTR haplotypes were identified, 10 in the PTC group and 13 in the control group, of which UTR-02 and UTR-01 haplotypes were the most frequent in the whole group (28.85% and 22.95%, respectively). In the PTC group, the UTR-01 haplotype was overrepresented (19.48%) (OR = 1.4895, 95% CI = 1.0180–2.1793; *p* = 0.0433), whereas the UTR-03 haplotype was underrepresented (OR = 0.4917, 95% CI = 0.3016–0.8015, *p* = 0.0041) when compared to the control group.

We identified 50 different *HLA-G* extended haplotypes, 32 among the PTC patients and 40 among the controls. The *G*010101a_(5′URR)_G*01:01:01:01_(coding)_UTR-01_(3’UTR)_ haplotype was the most frequent (19.51%), followed by *G*010102a_(5′URR)_G*01:01:02:01_(coding)_UTR-02_(3’UTR)_ (16.23%), considering the total studied group. Compared to the controls, the *G*0104a_(5′URR)_G*01:04:04_(coding)_UTR-03_(3’UTR)_ haplotype was underrepresented in the PTC patients (OR = 0.4488; 95% CI = 0.2221–0.9068; *p* = 0.0285).

### 2.2. HLA-G Gene Variability and Histopathological Features of PTC

To assess whether the *HLA-G* gene variability is associated with histopathological features related to PTC severity and prognosis, we analyzed the whole group of 185 PTC patients from two different geographical regions of Brazil (Table S2 for the *HLA-G* gene segments individually; Table 2 and Table S3 for the *HLA-G* extended haplotypes).

Considering the *HLA-G* 5′URR, the *G*0104a haplotype was underrepresented among patients presenting tumor multicentricity (OR = 0.3360, 95% CI = 0.1446–0.7810, *p* = 0.0089) compared to patients without this manifestation. The G*01:01:02:01 and G*01:04:01 coding region haplotypes were more frequently associated with poor incomplete response to therapy (OR = 2.0732; 95% CI = 1.0646–4.0374; *p* = 0.0392 and OR = 3.2484; 95% CI = 1.1552–9.1345; *p* = 0.0279, respectively). At the *HLA-G* 3′UTR segment, the frequency of UTR-02 was increased among patients presenting multicentricity (OR = 1.6759, 95% CI = 1.0616–2.6456, *p* = 0.0328), whereas the haplotype UTR-03 was less frequent (OR = 0.4106, 95% CI = 0.1912–0.8815, *p* = 0.0200) in these patients. Finally, the G0104a_(5′URR)_G*01:04:01_(coding)_UTR-03_(3′UTR)_ extended haplotype was associated with the absence of multifocal (OR = 0.2842, 95% CI = 0.0821–0.9836, *p* = 0.0362) and metastatic tumors (OR = 0.2520, 95% CI = 0.0577–1.1015, *p* = 0.0492); i.e., this extended haplotype protected against these features.

### 2.3. HLA-G Gene Variability and HLA-G Tumor Expression in PTC

We performed three previous studies concerning HLA-G in PTC: (i) a retrospective study evaluating only the HLA-G tissue expression in a different cohort of patients exhibiting several thyroidal disorders, including PTC (n = 72) [12], (ii) a retrospective study evaluating only the *HLA-G* 3′UTR segment in the same cohort of patients with different thyroidal disorders [35], and finally (iii) a prospective study using a new enlarged cohort of patients (n = 183) to assess HLA-G together with BRAF^V600E^ and *TERT* promoter mutations in PTC outcomes [11], markers individually associated with unfavorable tumor features and accelerated progression, remarkably in co-existence [7,11]. In the present study, we took advantage of the previous prospective study regarding HLA-G tissue expression in an increased number of PTC specimens to revisit the influence of the *HLA-G* gene variability (5′URR, coding, 3′UTR, and 5′URR/coding/3′UTR extended haplotypes) on the magnitude of the encoded protein expression (n = 170) (Table 3 and Table S4).

The *HLA-G* 5′URR *G*010101c haplotype was overrepresented in tumors presenting high HLA-G expression (>50% of positive tumor cells in PTC specimens) (OR = 6.3125, 95% CI = 0.8189–48.6592, *p* = 0.0457), while the coding region G*01:01:02:01 haplotype was associated with low HLA-G expression (≤50%) (OR = 0.4783, 95% CI = 0.2752–0.8311, *p* = 0.0111). Considering the extended haplotypes, increased HLA-G tumor expression was associated with the *G*010101c_(5′URR)_G*01:01:01:05_(coding)_UTR-04_(3′UTR)_ haplotype (OR = 6.3125, 95% CI = 0.8189–48.6592, *p* = 0.0457), while the *G*010102a_(5′URR)_G*01:01:02:01_(coding)_UTR-02_(3′UTR)_ haplotype was associated with decreased expression (OR = 0.5113, 95% CI = 0.2913–0.8974, *p* = 0.0230). However, we should emphasize that the associations with high HLA-G tissue expression (the *G*010101c haplotype and the extended *G*010101c_(5′URR)_G*01:01:01:05_(coding)_UTR-04_(3′UTR)_ haplotype) exhibited a very broad CI, including the number 1.

### 2.4. HLA-G Gene Variability and Soluble HLA-G Expression in PTC

A previous study was published evaluating plasma soluble HLA-G (sHLA-G) levels in PTC patients before and after thyroidectomy and the reciprocal influence of HLA-G and cytokines [36] by studying the same cohort of patients evaluated in this series. Thus, we also investigated the influence of the entire *HLA-G* gene variability on the sHLA-G plasma detection of PTC patients at the pre-thyroidectomy time (n = 84) (Table 4 and Table S5).

The 3′UTR UTR-18 haplotype was overrepresented in PTC patients exhibiting detectable plasma sHLA-G (OR = 12.6848, 95% CI = 0.7030–228.8785, *p* = 0.0295); however, it is worth mentioning the wide CI of this association, including the number 1. In contrast, the coding region G*01:01:02:01 haplotype (OR = 0.3658, 95% CI = 0.1546–0.8648, *p* = 0.0228), UTR-02 (OR = 0.4954, 95% CI = 0.2547–0.9636, *p* = 0.0453) and the G010102a_(5′URR)_G*01:01:02:01_(coding)_UTR-02_(3′UTR)_ extended haplotype (OR = 0.3658, 95% CI = 0.1546–0.8648, *p* = 0.0228) were associated with PTC exhibiting no detectable plasma sHLA-G at the pre-thyroidectomy time.

## 3. Discussion

Most TCs are differentiated tumors and, therefore, are associated with a low mortality rate [4]. However, 20–30% of these patients develop persistent or recurrent disease, most commonly in association with cervical and locoregional lymph nodes (LNs), requiring additional therapy and surgical intervention, which may increase morbidity [37]. The identification of patients presenting risk factors associated with aggressive disease can guide tumor management and prevent the overtreatment of patients with low-risk tumors [4]. Thus, many studies aiming to understand the role of genetic features in tumorigenesis and their effects on clinical DTC outcomes have so far been conducted to assess pre-operative diagnosis, prognosis, and treatment [38].

*HLA-G* gene polymorphisms have been studied in several types of human malignancies and have been associated with risk and predictive factors, and most of these studies are limited to evaluating single polymorphisms at the *HLA-G* regulatory gene segments (i.e., 5′URR and/or 3′UTR) [34]. Although many polymorphic sites along the *HLA-G* gene are in LD, a single polymorphism or a specific coding region haplotype may be associated with distinct regulatory region haplotypes [39], indicating that the typing of the entire gene is more adequate to investigate the *HLA-G* gene’s association with human tumors [23]. Indeed, in this study, we evaluated the *HLA-G* gene regions separately and as extended haplotypes in terms of their association with HLA-G tissue expression, sHLA-G plasma detection, and PTC outcomes.

The neo-expression of HLA-G in human tissues that constitutively do not express the molecule, as is the case of the thyroid, depends on the combination of factors present in the tumor milieu, such as environmental and hormonal factors, as well as genetic factors, including transcription and post-transcriptional factors [14]. These transcriptional and post-transcriptional factors may be differentially affected by the polymorphisms located at the 5′URR and 3′UTR of the *HLA-G* gene, respectively [9], yielding differential HLA-G expression in physiological and pathological conditions [40]. The *HLA-G* coding variability may affect the differential pattern of alternative splicing, leukocyte–receptor interaction, and peptide coupling [41,42]. We and other groups have previously reported that HLA-G is commonly expressed in PTC specimens [11,12], while sHLA-G plasma levels are frequently absent or detected at low levels [36,43]. The contrasting profile between the high HLA-G expression in the tumor microenvironment and the low plasma sHLA-G levels in PTC patients is still poorly understood. Both local modulatory factors and individual genetic features may account for the increased expression of HLA-G in the tumor microenvironment.

Undoubtedly, the *HLA-G* 3′UTR gene segment is the most studied one in terms of disease susceptibility and morbidity, particularly with respect to the presence (insertion or “Ins”) or absence (deletion or “Del”) of a 14-base-pair (14-bp) fragment, in which the 14-bp Del has been associated with increased HLA-G expression, whereas the 14-bp Ins has been associated with decreased HLA-G expression [44]. In this series, we reported that the *HLA-G* UTR-01 haplotype was overrepresented in PTC patients compared to controls. Since UTR-01 contains the 14-bp Del and is associated with increased sHLA-G levels even in healthy individuals [45,46], patients carrying the UTR-01 haplotype may have an additional risk of PTC development. In contrast, the UTR-03 haplotype that also contains the 14-bp Del was associated with protection against PTC development, as well as the G0104a_(5′URR)_G*01:04:04_(coding)_UTR-03_(3’UTR)_ extended haplotype. Considering that the *HLA-G* 3′UTR and the 5′URR segments present several elements that have been functionally studied, exhibiting potential roles in HLA-G production [40,45], the evaluation of a single polymorphism inside a specific gene region or a single gene region may only explore partial content of the HLA-G and disease association.

With respect to the association of *HLA-G* variability with clinical features, we observed that PTC patients carrying the (i) 5′URR *G*0104a haplotype, (ii) 3′UTR UTR-03 haplotype, and (iii) *G*0104a_(5′URR)_G*01:04:01_(coding)_UTR-03_(3′UTR)_ extended haplotype exhibited clinical benefit with respect to PTC development, since their frequencies were underrepresented in patients exhibiting metastatic and multifocal tumors. Cervical lymph node metastases (LNMs) are the main way through which PTC spread cancer cells [47] and present a worse prognosis due to a more frequent recurrence rate [48]. Tumor recurrence in the LNs after primary PTC treatment had an independent and significant negative effect on survival in patients aged over 45 years [47]. Although PTC metastasis is usually uncommon, it is associated with high mortality [49]. Nevertheless, the presence of neck LNM upon diagnosis was one of the most important factors associated with a greater risk of having distant metastases in a Brazilian cohort [50]. Additionally, multicentricity also represents a significant risk factor for disease progression in TC and increases the risk of disease recurrence [51]. In contrast, UTR-02 was associated with a risk of multicentricity, which is in agreement with our previous finding evaluating a smaller cohort of PTC patients [35].

Regarding the association between *HLA-G* gene variability with HLA-G tissue expression, we observed that the coding G*01:01:02:01 and the 3′UTR UTR-02 haplotypes individually or assembled as the *G*010102a_(5′URR)_G*01:01:02:01_(coding)_UTR-02_(3′UTR)_ extended haplotype exhibited an association with low HLA-G expression in PTC specimens. Individuals carrying UTR-02 have already been described as low sHLA-G producers [46]. In contrast, the 5′URR *G*010101c haplotype and the G010101c_(5′URR)_G*01:01:01:05_(coding)_UTR-04_(3′UTR)_ extended haplotype were associated with greater HLA-G expression in the PTC specimens. While UTR-04 has been associated with intermediate plasma sHLA-G levels in healthy Brazilian and French individuals [45], functional studies have reported that cells transfected with distinct *HLA-G* 5′URR yielded differential responses in the presence of HLA-G inducers or repressors. For instance, an HLA-G^+^ cell line (choriocarcinoma JEG-3) transfected with the *G*010101c haplotype significantly increased the luciferase activity cultured in the presence of interferon (IFN)-β when compared to non-treated transfectants [40]. Therefore, as mentioned previously, distinct *HLA-G* 5′URR, under the influence of diverse transcription factors may differentially induce gene expression. The balance between transcriptional (primarily acting at the 5′URR segment) and post-transcriptional (primarily acting at the 3′UTR segment) factors present in the PTC microenvironment may determine the magnitude of HLA-G tumor expression, corroborating the need to evaluate the entire gene variability.

Concerning sHLA-G detection, we observed that UTR-18 was associated with detectable sHLA-G, whereas the G010102a_(5′URR)_G*01:01:02:01_(coding)_UTR-02_(3′UTR)_ extended haplotype was associated with undetectable sHLA-G expression. The *HLA-G* UTR-02 haplotype is composed of the 14-bp Ins and +3142G; both variations were previously associated with low mRNA availability and decreased HLA-G expression, whereas the UTR-18 haplotype is composed of the 14-bp Del and +3142C associated with high HLA-G expression [45,52]. Additionally, an HLA-G^+^ cell line (melanoma FON^+^) transfected with the *G*010102a haplotype exhibited the lowest promoter activity among all other 5′URR haplotypes when compared to the empty vector [40].

A recent paper published by our research group characterized the entire *HLA-G* genetic variability in 4640 individuals from 88 different population samples across the globe from four datasets [53]. The study detected 442 different variants encompassing approximately 7870 nucleotides surrounding the *HLA-G locus*. These variants are arranged into 514 different coding haplotypes or full-length sequences. According to the study, in terms of global diversity, there are 14 *HLA-G* alleles with global frequencies higher than 1%: G*01:01:01:01, G*01:01:01:04, G*01:01:01:05, G*01:01:01:06, G*01:01:01:08, G*01:01:02:01, G*01:01:03:03, and G*01:01:22:01 (all of them encoding the G*01:01 protein), G*01:03:01:02 (encoding the G*01:03 protein), G*01:04:01:01, G*01:04:01:02, and G*01:04:04 (encoding the G*01:04 protein), G*01:05N (the null allele), and G*01:06:01:01 (encoding the G*01:06 protein). These alleles represent 91% of all HLA-G sequences detected worldwide, and their frequencies differ in each biogeographic region [53]. All of these *HLA-G* alleles were found in the present study and represent 96.9% of all detected haplotypes among the Brazilian subjects from the same geographical region.

In our study, three isolated coding region haplotypes were also associated with PTC: (i) the G*01:04:04 haplotype encodes the HLA-G*01:04 molecule and was associated with protection against disease development, (ii) the G*01:01:02:01 (HLA-G*01:01 molecule) and the G*01:04:01 (HLA-G*01:04 molecule) haplotypes were associated with poor biochemical or structural evidence of persistent disease, and (iii) the G*01:01:02:01 (HLA-G*01:01 molecule) haplotype was associated with undetectable sHLA-G. Although the HLA-G molecule may couple peptides in its groove, its major role is the modulation of immune responses through its binding to leukocyte receptors located outside the peptide-binding groove, whose interactive residues are conserved among these molecules [54]. The presence of a few proteins frequently observed worldwide corroborates the role of HLA-G as an immune checkpoint molecule rather than as an antigen-presenting molecule [53]. Thus, three major reasons may account for these associations: (i) a hitchhiking effect due to the LD between *HLA-G* coding and regulatory regions, (ii) different coding region haplotypes may generate differential alternative splicing mechanisms, yielding distinct HLA-G isoforms, and (iii) different coding region haplotypes (e.g., the G*01:04:01 and G*01:04:04) encoding the same molecule (HLA-G*01:04) may present distinct regulatory region haplotypes [39].

One limitation of the present study is that a few associations exhibited a very broad 95% CI and/or exhibited a 95% CI that includes the number 1, particularly involving less frequent *HLA-G* haplotypes. In human genetic studies, sample size plays a critical role in conducting reliable analyses of polymorphisms [55]. Thus, the OR and its associated 95% CI are valuable statistical measures to assess the strength and accuracy of associations between variables. These associations suggest that, for those haplotypes, the sample size of the study is not sufficient to produce a tight 95% CI and an OR measure that is truthfully estimated. Consequently, obtaining a sufficiently larger sample size would be necessary for more precise estimations. On the other hand, most of the significant associations presented a narrow 95% CI, a finding that suggests that our sample size is sufficient to produce statistically significant OR measures, supporting the reliability of the present results.

In contrast, using a Brazilian population is an important strength of our study for its remarkable and unique genetic diversity, resulting from miscegenation among Brazilian Native Amerindian, African, and European populations [56]. Genetic studies in a diverse population can uncover unique risk or protective variants that might not be apparent in more homogeneous populations. This knowledge is essential for advancing population medicine and/or personalized medicine, including but not limited to: (i) risk prediction, (ii) disease susceptibility, diagnosis, or prognosis, (iii) drug therapy optimization to increase treatment effectiveness and minimize adverse effects, and (iv) public health strategies for disease prevention and health promotion [57,58]. It is also important to emphasize the regional variations within Brazil. There are ancestry differences between regions since the degree of European, African, and Amerindian contributions are not homogeneous [59], which can have implications for genetic studies. Taking these geographical variations into account improves the relevance and accuracy of our findings since the risk association analysis for PTC development was performed evaluating only a specific Brazilian region.

## 4. Materials and Methods

### 4.1. Studied Population

The study was conducted on 185 patients with PTC (age at diagnosis 47.2 ± 15.8 years old; 154 female = 83.2%) submitted to thyroidectomy due to or the suspicion of PTC following the ultrasound-guided fine-needle aspiration (FNA) malignant cytology of the thyroid nodule (V and VI categories; 2017 Bethesda system for reporting thyroid cytopathology) [60]. A total of 34 cases were recruited from the “Hospital Liga Norte Riograndense Contra o Câncer”, Natal-RN, Brazil, and 151 were recruited from “Hospital das Clínicas de Ribeirão Preto”, Ribeirão Preto-SP, Brazil. The patients were followed up during a median period of 67 months (interquartile range, 45.7 to 125.3 months). Disease evolution during the follow-up was monitored by clinical examination, ultrasound imaging, and biochemical measurement of serum thyroglobulin (Tg) and anti-Tg antibody (TgAb) levels.

The cytological and histopathological analyses were performed by experienced pathologists, as well as the tumor node metastasis (TNM) staging classification according to the eighth edition of the American Joint Committee on Cancer (AJCC)/Union for International Cancer Control (UICC), ranging from stages I to IV to predict the risk of mortality from DTC. Overall, the lower the TNM stage, the lesser probability of cancer spread and the lower the risk of mortality [61].

Clinical and histopathological data of all patients (i.e., age at diagnosis, tumor size, histological subtype, tumor invasion, metastasis at diagnosis, multicentricity, extrathyroidal extension, Hashimoto’s thyroiditis, and TNM staging) as well as treatment details, including surgery approach and RAI therapy), are summarized in Table 5.

The individual response to initial conventional therapy of each patient at the last follow-up was assessed according to the criteria proposed by Momesso and Tuttle 2014 [62], adopted by the 2015 American Thyroid Association (ATA) guidelines [4], taking into account patients treated with thyroid lobectomy or total thyroidectomy, accompanied or not by RAI ablation. This assessment is a risk stratification system to predict recurrence/persistence of the disease based on four categories: (i) excellent response (no clinical, biochemical, or structural/imaging evidence of disease), (ii) indeterminate response (nonspecific biochemical or structural findings that require continuous observation), (iii) incomplete biochemical response (abnormal Tg or rising TgAb levels in the absence of localizable disease), and (iv) incomplete structural response (persistent or newly identified loco-regional or distant metastases, exhibiting abnormal Tg or TgAb or not).

A group of 154 unrelated healthy blood donors (aged 34 ± 11; 84 women = 54.6%) with no personal or family history of thyroid diseases and exhibiting negative anti-peroxidase antibodies was recruited from “Hospital das Clínicas de Ribeirão Preto”, Ribeirão Preto-SP, Brazil.

All subjects gave written informed consent to participate in the study, which was approved by local human research ethics committees (processes #18022/2014, and #029/029/2015).

### 4.2. HLA-G Genotyping

Genomic DNA was extracted from peripheral blood leucocytes using a standard salting out procedure [63]. The *HLA-G* 5′URR region was amplified by polymerase chain reaction (PCR) using the forward GPROMO.S 5′-ACATTCTAGAAGCTTCACAAGAATG and reverse GPROMO.R 5′-GCCTTGGTGTTCCGTGTCT primers, as previously described [22]. The sequencing was performed using the G-908R 5′-TTCACCTCACAGTTGTAAGTGTTC, G-830F 5′-CACACGGAAACTTAGGGCTACG, G-304R 5′-GCCAAGCGTTCTGTCTCAGTGT, and GPR-247 5′-CTCAAGCGTGGCTCTCAGGGTC primers.

The *HLA-G* coding region was PCR-amplified using the forward 5′-TAAAGTCCTCGCTCACCCAC and reverse 5′-CACCACCGACCCTGTTAAAG primers, as previously described [42,64], encompassing exon 1 to exon 4 (including introns). The PCR products were directly sequenced using 5′-TAAAGTCCTCGCTCACCCAC, 5′-GGGAAGAGGAGACACGGA, 5′-GGCCTCATAGTCAAAGACAGG, and 5′-CACCACCGACCCTGTTAAAG primers.

The end of the coding region, which covers exons 5 and 6, was amplified along with the 3′UTR segment. DNA amplification was performed using the forward 5′-CCATGAGAGATGCAAAGTGCT and reverse 5′- TCTTCTGATAACACAGGAACTTC primers [64]. The sequencing was performed using the 5′-CCATGAGAGATGCAAAGTGCT, 5′-CCTTTAACAGGGTCGGTGGTGAGG, 5′-TCTTCTGATAACACAGGAACTTC, and 5′-GATTGAAAAGGAGGGAGCT primers.

The sequences were analyzed and compared to the reference sequence recognized by the IMGT, <http://www.ebi.ac.uk/ipd/imgt/hla/> URL (accessed on 10 November 2020). All polymorphisms detected were individually annotated, and haplotypes of each *HLA-G* region were initially defined. These haplotypes were further used to construct *HLA-G* extended haplotypes, as previously described [39,53].

### 4.3. HLA-G Expression

The HLA-G expression in the tumor microenvironment of the PTC specimens was evaluated in the same series of patients using an immunohistochemical assay as previously described [11]. In short, we used the primary monoclonal antibody (mAb) anti-HLA-G MEM-G/2 (EXBIO, Vestec, Czech Republic) that recognizes all soluble and membrane-bound HLA-G molecules, diluted at 1:100 [64]. From the 185 PTC patients whose DNA was sequenced for *HLA-G* gene variability in the present study, the HLA-G expression in the tumor was available for 170. According to the percentage of stained tumor cells, HLA-G expression was stratified into two categories: low expression (≤50% of positive tumor cells in the PTC specimens) and high expression (>50% of positive tumor cells in the PTC specimens).

Plasma sHLA-G levels were previously evaluated in the same series of PTC patients before thyroidectomy to evaluate its relationship with several cytokines [36]. We detected sHLA-G levels by using a specific sandwich ELISA [45], using MEM-G/9 mouse anti-human HLA-G mAb (1:100, EXBIO, Praha, Czech Republic) as the capture antibody and rabbit anti-human β2-microglobulin (1:10.000, DAKO, Santa Clara, CA, USA) as the detection antibody. From the 185 PTC studied patients, soluble HLA-G levels were available for 84. Samples with sHLA-G levels below the detection limit were conventionally expressed as 0 ng/mL and categorically stratified into two categories: undetectable (0 ng/mL) or detectable sHLA-G levels.

### 4.4. Statistical Analysis

The LD among polymorphic sites at the *HLA-G* gene was evaluated by Haploview^®^ 4.2 the program [65]. Given the positive LD between the polymorphic sites, but unknown gametic phase, the PHASE algorithm [66] was used to infer the most likely haplotypes for each sample. Adherences of genotypic proportions to expectations under the Hardy–Weinberg equilibrium were tested by the exact test of Guo and Thompson [67] using ARLEQUIN 3.5 [68]. A two-tailed Fisher’s exact test was used for all comparisons, and OR and 95% CI were estimated. A 5% level of significance (α = 0.05) was considered for rejection of the null hypothesis.

## 5. Conclusions

The immune checkpoint HLA-G molecule is highly expressed in PTC specimens, although the impact of the *HLA-G* gene variability on PTC morbidity and/or the regulation of HLA-G expression in PTC was not fully understood until now. Therefore, by evaluating the entire *HLA-G* gene in PTC patients together with HLA-G expression in tumor specimens and sHLA-G plasma detection, we uncovered important associations between the *HLA-G* gene, taken as gene segments and/or as extended haplotypes, and: (i) the susceptibility to PTC development, (ii) tumor morbidity, particularly metastatic or multifocal tumors and poor response to conventional therapy, (iii) the magnitude of HLA-G expression in tumor specimens, and (iv) the presence or absence of detectable sHLA-G in pre-thyroidectomy plasma. Although several local and systemic (cytokines, hormones, hypoxia, etc.) factors contribute to both PTC development and HLA-G expression modulation, the associations reported in the present study can have significant implications in the context of PTC outcomes, particularly the identification of specific genetic markers that can serve as potential targets for personalized therapies in the future.

## Figures and Tables

**Table 1:** *HLA-G* extended haplotypes in papillary thyroid carcinoma (PTC) patients and control subjects.

*HLA-G* Extended Haplotypes				Total *n* = 305		Controls *n* = 154		PTC Patients *n* = 151		OR	95% CI	*p*
	5′URR	Coding	3′UTR	*n*	Freq.	*n*	Freq.	*n*	Freq.			
1	G010101a	G*01:01:01:01	UTR-01	119	0.1951	53	0.1721	66	0.2185	1.3455	0.8997–2.0124	0.1538
2	G010102a	G*01:01:02:01	UTR-02	99	0.1623	43	0.1396	56	0.1854	1.4029	0.9093–2.1645	0.1532
3	G0104a	G*01:04:01	UTR-03	39	0.0639	24	0.0779	15	0.0497	0.6185	0.3179–1.2034	0.1858
**4**	**G0104a**	G*01:04:04	**UTR-03**	**38**	**0.0623**	**26**	**0.0844**	**12**	**0.0397**	**0.4488**	**0.2221–0.9068**	**0.0285**
5	G010101b	G*01:01:01:05	UTR-04	36	0.0590	18	0.0584	18	0.0596	1.0211	0.5207–2.0025	1.0000
6	G010101c	G*01:01:01:05	UTR-04	33	0.0541	18	0.0584	15	0.0497	0.8420	0.4163–1.7031	0.7214
7	G010102a	G*01:06	UTR-02	31	0.0508	19	0.0617	12	0.0397	0.6294	0.3000–1.3203	0.2692
8	G010102a	G*01:05N	UTR-02	26	0.0426	12	0.0390	14	0.0464	1.1991	0.5453–2.6366	0.6924
9	G0103a	G*01:03:01:02	UTR-05	25	0.0410	12	0.0390	13	0.0430	1.1096	0.4980–2.4723	0.8405
10	G010102a	G*01:01:03:03	UTR-07	25	0.0410	13	0.0422	12	0.0397	0.9390	0.4214–2.0922	1.0000
11	G010101f	G*01:01:01:04	UTR-18	18	0.0295	9	0.0292	9	0.0298	1.0205	0.3995–2.6069	1.0000
12	G010101f	G*01:01:01:04	UTR-06	17	0.0279	6	0.0195	11	0.0364	1.9026	0.6946–5.2117	0.2273
13	G0103d	G*01:03:01:02	UTR-05	16	0.0262	9	0.0292	7	0.0232	0.7883	0.2898–2.1445	0.8012
14	G010101d	G*01:01:01:08	UTR-01	12	0.0197	4	0.0130	8	0.0265	2.0680	0.6161–6.9413	0.2579
15	G0103e	G*01:03:01:02	UTR-05	11	0.0180	4	0.0097	7	0.0232	1.8034	0.5225–6.2248	0.3791
16	G010102a	G*01:01:22:01	UTR-02	9	0.0148	4	0.0130	5	0.0166	1.2795	0.3403–4.8110	0.7499
17	G010101d	G*01:01:01:15	UTR-01	4	0.0066	1	0.0032	3	0.0099	3.0803	0.3186–29.7784	0.3689
18	G010102a	G*01:01:17	UTR-02	3	0.0049	1	0.0032	2	0.0066	2.0467	0.1846–22.6903	0.6207
19	G0104b	G*01:04:01	UTR-03	3	0.0049	2	0.0065	1	0.0033	0.5083	0.0458–5.6353	1.0000
20	G010102d	G*01:01:02:01	UTR-02	3	0.0049	1	0.0032	2	0.0066	2.0467	0.1846–22.6903	0.6207
21	G010101f	G*01:01:01:04	UTR-20	3	0.0049	2	0.0065	1	0.0033	0.5083	0.0458–5.6353	1.0000
22	G010101b	G*01:01:15^compatible^	UTR-06	2	0.0033	0	0.0000	2	0.0066	5.1331	0.2454–107.3643	0.2447
23	G010101f	G*01:01:01:13	UTR-06	2	0.0033	0	0.0000	2	0.0066	5.1331	0.2454–107.3643	0.2447
24	G010101a	G*01:01:01:01	UTR-06	2	0.0033	1	0.0032	1	0.0033	1.0199	0.0635–16.3806	1.0000
25	G010101a	G*01:01:01:06	UTR-04	2	0.0033	2	0.0065	0	0.0000	0.2026	0.0097–4.2385	0.4992
26	G0103c	G*01:03:01:02	UTR-05	2	0.0033	2	0.0065	0	0.0000	0.2026	0.0097–4.2385	0.4992
27	G0103e	G*01:03:01:02	UTR-17	2	0.0033	2	0.0065	0	0.0000	0.2026	0.0097–4.2385	0.4992
28	G010102a	G*01:01:14	UTR-02	2	0.0033	2	0.0065	0	0.0000	0.2026	0.0097–4.2385	0.4992
29	G010101a	G*01:01:01:04	UTR-06	2	0.0033	2	0.0065	0	0.0000	0.2026	0.0097–4.2385	0.4992
30	G010102a	G*01:01:02:01	UTR-08	2	0.0033	2	0.0065	0	0.0000	0.2026	0.0097–4.2385	0.4992
31	G010101h	G*01:01:09^(no deletion at +615)^	UTR-04	2	0.0033	2	0.0065	0	0.0000	0.2026	0.0097–4.2385	0.4992
32	G0103e	G*01:03:01:02	UTR-09	2	0.0033	2	0.0065	0	0.0000	0.2026	0.0097–4.2385	0.4992
33	G0104a	G*01:04:01	UTR-13	1	0.0016	0	0.0000	1	0.0033	3.0697	0.1246–75.6491	0.4951
34	G010101g	G*01:01:01:01	UTR-01	1	0.0016	0	0.0000	1	0.0033	3.0697	0.1246–75.6491	0.4951
35	G010102a	G*01:01:02:01^(+2798G)^	UTR-02	1	0.0016	0	0.0000	1	0.0033	3.0697	0.1246–75.6491	0.4951
36	G010101a	G*01:01:02:01	UTR-02	1	0.0016	0	0.0000	1	0.0033	3.0697	0.1246–75.6491	0.4951
37	G010101a	G*01:01:01:04	UTR-18	1	0.0016	0	0.0000	1	0.0033	3.0697	0.1246–75.6491	0.4951
38	G010101j	G*01:01:01:01	UTR-01	1	0.0016	0	0.0000	1	0.0033	3.0697	0.1246–75.6491	0.4951
39	G010101a	G*01:01:01:01	UTR-04	1	0.0016	0	0.0000	1	0.0033	3.0697	0.1246–75.6491	0.4951
40	G010101b	G*01:01:01:05	UTR-01	1	0.0016	0	0.0000	1	0.0033	3.0697	0.1246–75.6491	0.4951
41	G010101b	G*01:01:01:01	UTR-01	1	0.0016	1	0.0032	0	0.0000	0.3388	0.0137–8.3505	1.0000
42	G010101b	G*01:01:01:05	UTR-06	1	0.0016	1	0.0032	0	0.0000	0.3388	0.0137–8.3505	1.0000
43	G010101i	G*01:01:01:01	UTR-01	1	0.0016	1	0.0032	0	0.0000	0.3388	0.0137–8.3505	1.0000
44	G010102a	G*01:01:02:02	UTR-02	1	0.0016	1	0.0032	0	0.0000	0.3388	0.0137–8.3505	1.0000
45	G010102e	G*01:01:30:02	UTR-44	1	0.0016	1	0.0032	0	0.0000	0.3388	0.0137–8.3505	1.0000
46	G0103a	G*01:03:01:02	UTR-09	1	0.0016	1	0.0032	0	0.0000	0.3388	0.0137–8.3505	1.0000
47	G010102a	G*01:06	UTR-08	1	0.0016	1	0.0032	0	0.0000	0.3388	0.0137–8.3505	1.0000
48	G010101a	G*01:01:01:05	UTR-04	1	0.0016	1	0.0032	0	0.0000	0.3388	0.0137–8.3505	1.0000
49	G010102a	G*01:05N	UTR-08	1	0.0016	1	0.0032	0	0.0000	0.3388	0.0137–8.3505	1.0000
50	G0104c	G*01:04:05^compatible^	UTR-03	1	0.0016	1	0.0032	0	0.0000	0.3388	0.0137–8.3505	1.0000
	**Total [2*n*]**			**610**	**1.0000**	**308**	**1.0000**	**302**	**1.0000**	**-**	**-**	**-**

5′URR: 5′ upstream region. 3′UTR: 3′ untranslated region. OR: odds ratio. CI: confidence interval. Freq: frequency. Haplotypes in order of total frequency. Comparisons between PTC patients and controls were made by using Fisher’s exact test, and *p*-values below 0.05 are highlighted in bold.

**Table 2:** Association between *HLA-G* extended haplotypes and histopathological features of PTC (*n* = 185).

*HLA-G* Extended Haplotypes				Tumor Size		Histological Subtype ϕ		Tumor Invasion		Extrathyroidal Extension		Multicentricity		Metastasis at Diagnosis		TNM Staging 8th θ		Hashimoto’s Thyroiditis		Response to Therapy #	
	5′URR	Coding	3′UTR	<2 cm *n* = 106	≥2 cm *n* = 79	Low Risk *n* = 152	High Risk *n* = 33	Absent *n* = 133	Present *n* = 52	Absent *n* = 134	Present *n* = 51	Absent *n* = 119	Present *n* = 66	Absent *n* = 133	Present *n* = 52	I *n* = 153	II/III/IV *n* = 30	Absent *n* = 132	Present *n* = 53	Complete *n* = 111	Incomplete *n* = 29
				*n* (freq.)	*n* (freq.)	*n* (freq.)	*n* (freq.)	*n* (freq.)	*n* (freq.)	*n* (freq.)	*n* (freq.)	*n* (freq.)	*n* (freq.)	*n* (freq.)	*n* (freq.)	*n* (freq.)	*n* (freq.)	*n* (freq.)	*n* (freq.)	*n* (freq.)	*n* (freq.)
1	G010101a	G*01:01:01:01	UTR-01	46 (9.2170)	32 (0.2025)	62 (0.2039)	16 (0.2424)	55 (0.2068)	23 (0.2212)	57 (0.2127)	21 (0.2059)	52 (0.2185)	26 (0.1970)	58 (0.2180)	20 (0.1923)	67 (0.2189)	11 (0.1833)	51 (0.1931)	27 (0.2547)	49 (0.2207)	9 (0.1551)
2	G010102a	G*01:01:02:01	UTR-02	39 (0.1840)	28 (0.1772)	54 (0.1776)	13 (0.1969)	48 (0.1805)	19 (0.1827)	50 (0.1866)	17 (0.1667)	40 (0.1681)	27 (0.2045)	47 (0.1767)	20 (0.1923)	53 (0.1732)	13 (0.2166)	52 (0.1969)	15 (0.1415)	37 (0.1666)	16 (0.2758)
**3**	**G0104a**	G*01:04:01	**UTR-03**	10 (0.0472)	11 (0.0696)	17 (0.0559)	4 (0.0606)	16 (0.0602)	5 (0.0481)	15 (0.0560)	6 (0.0588)	**18 ^A^** **(0.0756)**	**3 ^A^** **(0.0227)**	**19 ^B^** **(0.0714)**	**2 ^B^** **(0.0192)**	17 (0.0555)	4 (0.0666)	16 (0.0606)	5 (0.0471)	9 (0.0405)	5 (0.0862)
4	G0104a	G*01:04:04	UTR-03	8 (0.0377)	11 (0.0696)	17 (0.0559)	2 (0.0303)	15 (0.0564)	4 (0.0385)	12 (0.0448)	7 (0.0686)	15 (0.0630)	4 (0.0303)	13 (0.0489)	6 (0.0577)	13 (0.0424)	4 (0.0666)	16 (0.0606)	3 (0.0283)	10 (0.0450)	1 (0.0172)
5	G010101b	G*01:01:01:05	UTR-04	10 (0.0472)	9 (0.0570)	15 (0.0493)	4 (0.0606)	13 (0.0489)	6 (0.0577)	15 (0.0560)	4 (0.0392)	10 (0.0420)	9 (0.0682)	16 (0.0602)	3 (0.0288)	18 (0.0588)	1 (0.0166)	15 (0.0568)	4 (0.0377)	13 (0.0585)	3 (0.0517)
6	G0103a	G*01:03:01:02	UTR-05	14 (0.0660)	4 (0.0253)	15 (0.0493)	3 (0.0454)	13 (0.0489)	5 (0.0481)	13 (0.0485)	5 (0.0490)	13 (0.0546)	5 (0.0379)	14 (0.0526)	4 (0.0385)	13 (0.0424)	5 (0.0833)	9 (0.0340)	9 (0.0849)	10 (0.0450)	3 (0.0517)
7	G010101c	G*01:01:01:05	UTR-04	8 (0.0377)	9 (0.0570)	13 (0.0427)	4 (0.0606)	15 (0.0564)	2 (0.0192)	15 (0.0560)	2 (0.0196)	13 (0.0546)	4 (0.0303)	11 (0.0414)	6 (0.0577)	15 (0.0490)	2 (0.0333)	10 (0.0378)	7 (0.0660)	13 (0.0585)	2 (0.0344)
8	G010102a	G*01:05N	UTR-02	10 (0.0472)	6 (0.0380)	14 (0.0460)	2 (0.0303)	12 (0.0451)	4 (0.0385)	12 (0.0448)	4 (0.0392)	10 (0.0420)	6 (0.0455)	12 (0.0451)	4 (0.0385)	15 (0.0490)	1 (0.0166)	11 (0.0416)	5 (0.0471)	11 (0.0495)	2 (0.0344)
9	G010102a	G*01:06	UTR-02	11 (0.0519)	4 (0.0253)	11 (0.0361)	4 (0.0606)	12 (0.0451)	3 (0.0288)	11 (0.0410)	4 (0.0392)	8 (0.0336)	7 (0.0530)	11 (0.0414)	4 (0.0385)	14 (0.0457)	1 (0.0166)	9 (0.0340)	6 (0.0566)	10 (0.0450)	1 (0.0172)
10	G010102a	G*01:01:03:03	UTR-07	8 (0.0377)	6 (0.0380)	11 (0.0361)	3 (0.0454)	10 (0.0376)	4 (0.0385)	11 (0.0410)	3 (0.0294)	10 (0.0420)	4 (0.0303)	8 (0.0301)	6 (0.0577)	11 (0.0359)	3 (0.0500)	12 (0.0454)	2 (0.0188)	10 (0.0450)	2 (0.0344)
11	G010101f	G*01:01:01:04	UTR-06	7 (0.0330)	5 (0.0316)	11 (0.0361)	1 (0.0151)	7 (0.0263)	5 (0.0481)	9 (0.0336)	3 (0.0294)	7 (0.0294)	5 (0.0379)	8 (0.0301)	4 (0.0385)	11 (0.0359)	1 (0.0166)	11 (0.0416)	1 (0.0094)	8 (0.0360)	2 (0.0344)
12	G010101f	G*01:01:01:04	UTR-18	6 (0.0283)	5 (0.0316)	10 (0.0328)	1 (0.0151)	7 (0.0263)	4 (0.0385)	8 (0.0299)	3 (0.0294)	6 (0.0252)	5 (0.0379)	7 (0.0263)	4 (0.0385)	7 (0.0229)	4 (0.0667)	7 (0.0265)	4 (0.0377)	8 (0.0360)	1 (0.0172)
13	G0103e	G*01:03:01:02	UTR-05	6 (0.0283)	4 (0.0253)	10 (0.0328)	0 (0.0000)	7 (0.0263)	3 (0.0288)	5 (0.0187)	5 (0.0490)	9 (0.0378)	1 (0.0076)	7 (0.0263)	3 (0.0288)	8 (0.0261)	2 (0.0333)	9 (0.0340)	1 (0.0094)	6 (0.0270)	0 (0.0000)
14	G010101d	G*01:01:01:08	UTR-01	6 (0.0283)	3 (0.0190)	9 (0.0296)	0 (0.0000)	5 (0.0188)	4 (0.0385)	6 (0.0224)	3 (0.0294)	6 (0.0252)	3 (0.0227)	6 (0.0226)	3 (0.0288)	8 (0.0261)	1 (0.0166)	9 (0.0340)	0 (0.0000)	6 (0.0270)	1 (0.0172)
15	G0103d	G*01:03:01:02	UTR-05	3 (0.0142)	4 (0.0253)	6 (0.0197)	1 (0.0151)	7 (0.0263)	0 (0.0000)	6 (0.0224)	1 (0.0098)	3 (0.0126)	4 (0.0303)	6 (0.0226)	1 (0.0096)	7 (0.0228)	0 (0.0000)	7 (0.0265)	0 (0.0000)	4 (0.0180)	3 (0.0517)
16	G010102a	G*01:01:22:01	UTR-02	6 (0.0283)	1 (0.0063)	6 (0.0197)	1 (0.0151)	5 (0.0188)	2 (0.0192)	5 (0.0187)	2 (0.0196)	1 (0.0042)	6 (0.0455)	4 (0.0150)	3 (0.0288)	6 (0.0196)	0 (0.0000)	2 (0.0075)	5 (0.0471)	3 (0.0135)	2 (0.0344)
17	G0104b	G*01:04:01	UTR-03	3 (0.0142)	2 (0.0127)	5 (0.0164)	0 (0.0000)	3 (0.0113)	2 (0.0192)	3 (0.0112)	2 (0.0196)	3 (0.0126)	2 (0.0152)	3 (0.0113)	2 (0.0192)	5 (0.0163)	0 (0.0000)	2 (0.0075)	3 (0.0283)	0 (0.0000)	1 (0.0172)
Others *				11 (0.0519)	14 (0.0885)	18 (0.0581)	7 (0.1058)	16 (0.0602)	9 (0.0864)	15 (0.0560)	10 (0.0980)	14 (0.0588)	11 (0.0836)	16 (0.0602)	9 (0.0864)	18 (0.0588)	7 (0.1164)	16 (0.0597)	9 (0.0846)	15 (0.0675)	4 (0.0688)

5′URR: 5′ upstream region. 3′UTR: 3′ untranslated region. Freq: frequency. Haplotypes in order of total frequency. * Details about the other less frequent *HLA-G* extended haplotypes are available at Table S3. Association of haplotypes with histopathological features of PTC was made by using Fisher’s exact test, and *p*-values below 0.05 are highlighted in bold. ϕ Low-risk/more frequent subtypes (classic + follicular) or high-risk/less frequent subtypes (oncocytic, solid, trabecular, and others). θ Information not available for 2 PTC patients (total *n* = 183). Tumor node metastasis (TNM) system refers to the primary tumor, lymph node metastasis, and distant metastasis. # Information not available for 45 PTC patients (total *n* = 140). Complete (the sum of patients presenting excellent and indeterminate responses) or incomplete (the sum of patients presenting biochemical and structural incomplete responses). **^A^** OR: 0.2842; 95% CI: 0.0821–0.9836; *p*: 0.0362. **^B^** OR: 0.2520; 95% CI: 0.0577–1.1015; *p*: 0.0492. OR: odds ratio. CI: confidence interval.

**Table 3:** *HLA-G* extended haplotypes in papillary thyroid carcinoma (PTC) patients according to the expression of HLA-G in the tumor microenvironment [n = 170].

*HLA-G* Extended Haplotypes					HLA-G Tumor Expression #						
					Low *n* = 51		High *n* = 119		OR	95% CI	*p*
	5′URR	Coding	3′UTR	*n*	*n*	Freq.	*n*	Freq.			
1	G010101a	G*01:01:01:01	UTR-01	71	19	0.1863	52	0.2185	1.2213	0.6799–2.1936	0.5620
**2**	**G010102a**	G*01:01:02:01	**UTR-02**	**64**	**27**	**0.2647**	**37**	**0.1555**	**0.5113**	**0.2913–0.8974**	**0.0230**
3	G0104a	G*01:04:04	UTR-03	19	6	0.0588	13	0.0546	0.9244	0.3413–2.5038	1.0000
4	G0104a	G*01:04:01	UTR-03	17	8	0.0784	9	0.0378	0.4618	0.1729–1.2330	0.1712
5	G010101b	G*01:01:01:05	UTR-04	16	6	0.0588	10	0.0420	0.7018	0.2480–1.9850	0.5774
6	G0103a	G*01:03:01:02	UTR-05	16	5	0.0490	11	0.0462	0.9401	0.3181–2.7780	1.0000
**7**	**G010101c**	G*01:01:01:05	**UTR-04**	**15**	**1**	**0.0098**	**14**	**0.0588**	**6.3125**	**0.8189–48.6592**	**0.0457**
8	G010102a	G*01:06	UTR-02	15	4	0.0392	11	0.0462	1.1872	0.3689–3.8200	1.0000
9	G010102a	G*01:05N	UTR-02	13	2	0.0196	11	0.0462	2.4229	0.5273–11.1323	0.3583
10	G010102a	G*01:01:03:03	UTR-07	13	2	0.0196	11	0.0462	2.4229	0.5273–11.1323	0.3583
11	G010101f	G*01:01:01:04	UTR-06	12	3	0.0294	9	0.0378	1.2969	0.3437–4.8927	1.0000
12	G010101f	G*01:01:01:04	UTR-18	11	3	0.0294	8	0.0336	1.1478	0.2982–4.4172	1.0000
13	G010101d	G*01:01:01:08	UTR-01	8	3	0.0294	5	0.0210	0.7082	0.1660–3.0205	0.7007
14	G0103e	G*01:03:01:02	UTR-05	8	2	0.0196	6	0.0252	1.2931	0.2565–6.5171	1.0000
15	G010102a	G*01:01:22:01	UTR-02	7	0	0.0000	7	0.0294	6.6415	0.3757–117.3865	0.1076
16	G0103d	G*01:03:01:02	UTR-05	6	1	0.0098	5	0.0210	2.1674	0.2500–18.7878	0.6729
17	G0104b	G*01:04:01	UTR-03	5	2	0.0196	3	0.0126	0.6383	0.1050–3.8786	0.6383
24	G010101d	G*01:01:01:15	UTR-01	4	0	0.0000	4	0.0168	3.9339	0.2098–73.7422	0.3207
18	G010101f	G*01:01:01:04	UTR-20	2	0	0.0000	2	0.0084	2.1670	0.1031–45.5380	1.0000
19	G010101a	G*01:01:01:01	UTR-06	2	1	0.0098	1	0.0042	0.4262	0.0263–6.8801	0.5106
20	G010102d	G*01:01:02:01	UTR-02	2	1	0.0098	1	0.0042	0.4262	0.0263–6.8801	0.5106
21	G010102a	G*01:01:17	UTR-02	2	1	0.0098	1	0.0042	0.4262	0.0263–6.8801	0.5106
22	G010101b	G*01:01:15^compatible^	UTR-06	2	0	0.0000	2	0.0084	2.1670	0.1031–45.5380	1.0000
23	G010101f	G*01:01:01:13	UTR-06	2	1	0.0098	1	0.0042	0.4262	0.0263–6.8801	0.5106
25	G0104a	G*01:04:01	UTR-13	1	1	0.0098	0	0.0000	0.1419	0.0057–3.5117	0.3000
26	G010101g	G*01:01:01:01	UTR-01	1	1	0.0098	0	0.0000	0.1419	0.0057–3.5117	0.3000
27	G010102a	G*01:01:02:01^(+2798G)^	UTR-02	1	1	0.0098	0	0.0000	0.1419	0.0057–3.5117	0.3000
28	G010101a	G*01:01:02:01	UTR-02	1	1	0.0098	0	0.0000	0.1419	0.0057–3.5117	0.3000
29	G010101j	G*01:01:01:01	UTR-01	1	0	0.0000	1	0.0042	1.2947	0.0523–32.0496	1.0000
30	G010101a	G*01:01:01:01	UTR-04	1	0	0.0000	1	0.0042	1.2947	0.0523–32.0496	1.0000
31	G010101b	G*01:01:01:05	UTR-01	1	0	0.0000	1	0.0042	1.2947	0.0523–32.0496	1.0000
32	G010101h	G*01:01:01:06	UTR-04	1	0	0.0000	1	0.0042	1.2947	0.0523–32.0496	1.0000
	**Total [2*n*]**			**340**	**102**	**1.0000**	**238**	**1.0000**	**-**	**-**	**-**

5′URR: 5′ upstream region. 3′UTR: 3′ untranslated region. OR: odds ratio. CI: confidence interval. Freq: frequency. Haplotypes in order of total frequency. Association of haplotypes with HLA-G tumor expression was made by using Fisher’s exact test, and *p*-values below 0.05 are highlighted in bold. # Low (≤50% of positive tumor cells) or high (>50% of positive tumor cells).

**Table 4:** *HLA-G* extended haplotypes in papillary thyroid carcinoma (PTC) patients according to sHLA-G detection in plasma before thyroidectomy [*n* = 84].

*HLA-G* Extended Haplotypes					sHLA-G						
					Undetectable *n* = 40		Detectable *n* = 44		OR	95% CI	*p*
	5′URR	Coding	3′UTR	*n*	*n*	Freq.	*n*	Freq.			
**1**	**G010102a**	G*01:01:02:01	**UTR-02**	**28**	**19**	**0.2375**	**9**	**0.1023**	**0.3658**	**0.1546–0.8648**	**0.0228**
2	G010101a	G*01:01:01:01	UTR-01	27	11	0.1375	16	0.1818	1.3939	0.6043–3.2149	0.5296
3	G0104a	G*01:04:01	UTR-03	10	3	0.0375	7	0.0795	2.2181	0.5535–8.8876	0.3345
4	G0104a	G*01:04:04	UTR-03	8	5	0.0625	3	0.0341	0.5294	0.1223–2.2902	0.4802
5	G010102a	G*01:06	UTR-02	8	6	0.0750	2	0.0227	0.2868	0.0561–1.4641	0.1528
6	G0103a	G*01:03:01:02	UTR-05	8	2	0.0250	6	0.0682	2.8537	0.5590–14.5654	0.2817
7	G010102a	G*01:05N	UTR-02	8	4	0.0500	4	0.0455	0.9048	0.2186–3.7439	1.0000
8	G010101f	G*01:01:01:04	UTR-06	8	5	0.0625	3	0.0341	0.5294	0.1223–2.2902	0.4802
9	G010101b	G*01:01:01:05	UTR-04	7	4	0.0500	3	0.0341	0.6706	0.1454–3.0925	0.7098
10	G010101c	G*01:01:01:05	UTR-04	7	3	0.0375	4	0.0455	1.2222	0.2650–5.6362	1.0000
11	G010102a	G*01:01:03:03	UTR-07	7	2	0.0250	5	0.0568	2.3494	0.4428–12.4641	0.4470
12	G0103e	G*01:03:01:02	UTR-05	7	3	0.0375	4	0.0455	1.2222	0.2650–5.6362	1.0000
13	G010101f	G*01:01:01:04	UTR-18	5	0	0.0000	5	0.0568	10.6048	0.5770–194.9039	0.0602
14	G010101d	G*01:01:01:08	UTR-01	5	3	0.0375	2	0.0227	0.5969	0.0971–3.6671	0.6698
15	G010102a	G*01:01:22:01	UTR-02	5	2	0.0250	3	0.0341	1.3765	0.2240–8.4563	1.0000
16	G0104b	G*01:04:01	UTR-03	5	3	0.0375	2	0.0227	0.5969	0.0971–3.6671	0.6698
17	G0103d	G*01:03:01:02	UTR-05	4	3	0.0375	1	0.0114	0.2950	0.0300–2.8954	0.3480
18	G010102a	G*01:01:17	UTR-02	2	0	0.0000	2	0.0227	4.6532	0.2200–98.4006	0.4981
19	G010101d	G*01:01:01:15	UTR-01	2	0	0.0000	2	0.0227	4.6532	0.2200–98.4006	0.4981
20	G010101f	G*01:01:01:04	UTR-20	1	1	0.0125	0	0.0000	0.2994	0.0120–7.4562	0.4762
21	G010101a	G*01:01:01:01	UTR-06	1	0	0.0000	1	0.0114	2.7600	0.1108–68.7254	1.0000
22	G010102a	G*01:01:02:01^(+2798G)^	UTR-02	1	0	0.0000	1	0.0114	2.7600	0.1108–68.7254	1.0000
23	G010101a	G*01:01:01:04	UTR-18	1	0	0.0000	1	0.0114	2.7600	0.1108–68.7254	1.0000
24	G010101a	G*01:01:01:01	UTR-04	1	1	0.0125	0	0.0000	0.2994	0.0120–7.4562	0.4762
25	G010101b	G*01:01:01:05	UTR-01	1	0	0.0000	1	0.0114	2.7600	0.1108–68.7254	1.0000
26	G010101h	G*01:01:01:06	UTR-04	1	0	0.0000	1	0.0114	2.7600	0.1108–68.7254	1.0000
	**Total [2*n*]**			**168**	**80**	**1.0000**	**88**	**1.0000**	**-**	**-**	**-**

5′URR: 5′ upstream region. 3′UTR: 3′ untranslated region. OR: odds ratio. CI: confidence interval. Freq: frequency. Haplotypes in order of total frequency. Association of haplotypes with sHLA-G detection was made by using Fisher’s exact test, and *p*-values below 0.05 are highlighted in bold.

**Table 5:** Clinical and histopathological features of the papillary thyroid carcinoma (PTC) patients.

	*n*	%
Surgical strategy		
Total thyroidectomy	182	98.4
Information not available	3	1.6
Radioactive iodine (RAI) ablation		
Yes	152	82.2
No	31	16.7
Information not available	2	1.1
Tumor size (cm) (mean: 2.14)		
<2	106	57.3
≥2	79	42.7
Histological subtype		
Classic	96	51.9
Follicular	44	23.8
Oncocytic	17	9.2
Classic and follicular	12	6.5
Follicular and oncocytic	5	2.7
Follicular and solid	4	2.2
Solid	3	1.6
Trabecular	2	1.1
Others	2	1.1
Tumor invasion		
Absent	133	71.9
Present:	52	28.1
Angiolymphatic	17	9.2
Vascular	11	5.9
Capsular	11	5.9
Capsular and vascular	3	1.6
Angiolymphatic and capsular	3	1.6
Angiolymphatic and perineural	3	1.6
Others	4	2.2
Extrathyroidal extension ^a^		
Absent	134	72.4
Present	51	27.6
Multicentricity		
Absent	119	64.3
Present	66	35.7
Metastasis at diagnosis		
Absent	133	71.9
Present:	52	28.1
Lymph node(s)	47	25.4
Lymph node(s) and lung	3	1.6
Lymph node(s), lung, and bone	1	0.5
Lung	1	0.5
Hashimoto’s thyroiditis		
Absent	132	71.4
Present	53	28.6
TNM Staging 8th ^b^		
I	153	82.7
II	24	13.0
III	3	1.6
IV	3	1.6
Information not available	2	1.1
Response to therapy ^c^		
Excellent	80	43.2
Indeterminate	31	16.8
Biochemical incomplete	13	7.0
Structural incomplete	16	8.6
Information not available	45	24.3

^a^ Including minimal (immediate perithyroidal soft tissues or sternothyroid muscle typically detected only microscopically) and extensive (subcutaneous soft tissues, i.e., larynx, trachea, esophagus, or recurrent laryngeal nerve) extrathyroidal extension. ^b^ At initial diagnosis. Tumor node metastasis (TNM) system refers to the primary tumor, lymph node metastasis, and distant metastasis. ^c^ Classification at the final follow-up.

## Data Availability

The data can be requested from the corresponding authors.

## Supplementary Materials

The following supporting information can be downloaded at: https://www.mdpi.com/article/10.3390/ijms241612858/s1.
