# Supplementary material for: HLA-G Gene Variability Is Associated with Papillary Thyroid Carcinoma Morbidity and the HLA-G Protein Profile
Source: Int J Mol Sci. 2023 Aug 16;24(16):12858. doi: 10.3390/ijms241612858 (PMC10454351; doi:10.3390/ijms241612858)
Supplement: Supplementary file 1 [file ijms-24-12858-s001.zip › ijms-2535544-supplementary.pdf]

SUPPLEMENTARY MATERIAL

Article

HLA-G Gene Variability Is Associated with Papillary Thyroid Carcinoma Morbidity and the HLA-G Protein Profile

Bruna C. Bertol [1,2,*], Guilherme Debortoli [3], Fabrício C. Dias [4], Jéssica N. G. de Araújo [5], Luana S. M. Maia [4], Bibiana S. de Almeida [4], Nathalie L. de Figueiredo-Feitosa [6], Luiz Carlos C. de Freitas [7], Erick C. Castelli [8], Celso T. Mendes-Junior [9], Vivian N. Silbiger [5], Léa M. Z. Maciel [6] and Eduardo A. Donadi [1,4,*]

1 Postgraduate Program of Basic and Applied Immunology, Ribeirão Preto Medical School, University of São Paulo, Ribeirão Preto 14049-900, Brazil
2 Princess Margaret Cancer Centre, University Health Network, Toronto, ON M5G 2M9, Canada
3 Department of Anthropology, University of Toronto at Mississauga, Mississauga, ON L5L 1C6, Canada; guilherme.debortoli@mail.utoronto.ca
4 Division of Clinical Immunology, Department of Medicine, Ribeirão Preto Medical School, University of São Paulo, Ribeirão Preto 14049-900, Brazil; diasfc@gmail.com (F.C.D.); luana.sellamottamaia@unibas.ch (L.S.M.M.); bibianasgorla@hotmail.com (B.S.d.A.)
5 Department of Clinical Analysis and Toxicology, Federal University of Rio Grande do Norte, Natal 59012-570, Brazil; jessnaysub@hotmail.com (J.N.G.d.A.); viviansilbiger@hotmail.com (V.N.S.)
6 Division of Endocrinology and Metabolism, Department of Medicine, Ribeirão Preto Medical School, University of São Paulo, Ribeirão Preto 14049-900, Brazil; nathaliefigueiredo@yahoo.com.br (N.L.d.F.-F.); leamaciel@hcrp.usp.br (L.M.Z.M.)
7 Department of Ophthalmology, Otorhinolaryngology and Head and Neck Surgery, Ribeirão Preto Medical School, University of São Paulo, Ribeirão Preto 14049-900, Brazil; lcconti@fmrp.usp.br
8 Department of Pathology, School of Medicine, São Paulo State University, Botucatu 18618-687, Brazil; erick.castelli@unesp.br
9 Departamento de Química, Faculdade de Filosofia, Ciências e Letras de Ribeirão Preto, Universidade de São Paulo, Ribeirão Preto 14049-900, Brazil; ctmendes@ffclrp.usp.br
* Correspondence: bruna.bertol@uhn.ca (B.C.B.); eadonadi@fmrpusp.br (E.A.D.); Tel.: +55-16-3602-2566 (E.A.D.)

Table S1. *HLA-G* 5′URR, coding and 3'UTR haplotypes in papillary thyroid carcinoma (PTC) patients and control subjects.

		HLA-G haplotypes	Total		Controls		PTC patients		OR	95% CI	*P*
			n	Freq.	*n*	Freq.	*n*	Freq.			
5′URR	1	G010102a	201	0.3295	99	0.3214	102	0.3377	1.0767	0.7681 - 1.5092	0.7305
	2	G010101a	129	0.2115	59	0.1916	70	0.2318	1.2734	0.8623 - 1.8804	0.2352
	**3**	**G0104a**	**78**	**0.1279**	**50**	**0.1623**	**28**	**0.0927**	**0.5273**	**0.3221 - 0.8632**	**0.0109**
	4	G010101b	41	0.0672	20	0.0649	21	0.0695	1.0762	0.5708 - 2.0288	0.8723
	5	G010101f	40	0.0656	17	0.0552	23	0.0762	1.4111	0.7381 - 2.6978	0.3288
	6	G010101c	33	0.0541	18	0.0584	15	0.0497	0.8420	0.4163 - 1.7031	0.7214
	7	G0103a	26	0.0426	13	0.0422	13	0.0430	1.0208	0.4653 - 2.2395	1.0000
	8	G010101d	16	0.0262	5	0.0162	11	0.0364	2.2907	0.7863 - 6.6736	0.1348
	9	G0103d	16	0.0262	9	0.0292	7	0.0232	0.7883	0.2898 - 2.1445	0.8012
	10	G0103e	15	0.0246	8	0.0260	7	0.0232	0.8898	0.3186 - 2.4851	1.0000
	11	G0104b	3	0.0049	2	0.0065	1	0.0033	0.5083	0.0458 - 5.6353	1.0000
	12	G010102d	3	0.0049	1	0.0032	2	0.0066	2.0467	0.1846 - 22.6903	0.6207
	13	G010101h	2	0.0033	2	0.0065	0	0.0000	0.2026	0.0097 - 4.2385	0.4992
	14	G0103c	2	0.0033	2	0.0065	0	0.0000	0.2026	0.0097 - 4.2385	0.4992
	15	G010101g	1	0.0016	0	0.0000	1	0.0033	3.0697	0.1246 - 75.6491	0.4951
	16	G010101j	1	0.0016	0	0.0000	1	0.0033	3.0697	0.1246 - 75.6491	0.4951
	17	G010101i	1	0.0016	1	0.0032	0	0.0000	0.3388	0.0137 - 8.3505	1.0000
	18	G010102e	1	0.0016	1	0.0032	0	0.0000	0.3388	0.0137 - 8.3505	1.0000
	19	G0104c	1	0.0016	1	0.0032	0	0.0000	0.3388	0.0137 - 8.3505	1.0000
		**Total [2*n*]**	**610**	**1.0000**	**308**	**1.0000**	**302**	**1.0000**	-	-	-
Coding	1	G*01:01:01:01	126	0.2066	56	0.1818	70	0.2318	1.3578	0.9156 - 2.0135	0.1345
	2	G*01:01:02:01	105	0.1721	46	0.1494	59	0.1954	1.3829	0.9057 - 2.1114	0.1350
	3	G*01:01:01:05	72	0.1180	38	0.1234	34	0.1126	0.9014	0.5508 - 1.4752	0.7079
	4	G*01:03:01:02	59	0.0967	32	0.1039	27	0.0894	0.8468	0.4941 - 1.4513	0.5852
	5	G*01:04:01	43	0.0705	26	0.0844	17	0.0563	0.6470	0.3435 - 1.2185	0.2063
	6	G*01:01:01:04	41	0.0672	19	0.0617	22	0.0728	1.1951	0.6330 - 2.2562	0.6296
	**7**	**G*01:04:04**	**38**	**0.0623**	**26**	**0.0844**	**12**	**0.0397**	**0.4488**	**0.2221 - 0.9068**	**0.0285**
	8	G*01:06	32	0.0525	20	0.0649	12	0.0397	0.5959	0.2860 - 1.2414	0.2038
	9	G*01:05N	27	0.0443	13	0.0422	14	0.0464	1.1031	0.5096 - 2.3876	0.8459
	10	G*01:01:03:03	25	0.0410	13	0.0422	12	0.0397	0.9390	0.4214 - 2.0922	1.0000
	11	G*01:01:01:08	12	0.0197	4	0.0130	8	0.0265	2.0680	0.6161 - 6.9413	0.2579
	12	G*01:01:22:01	9	0.0148	4	0.0130	5	0.0166	1.2795	0.3403 - 4.8110	0.7499
	13	G*01:01:01:15	4	0.0066	1	0.0032	3	0.0099	3.0803	0.3186 - 29.7784	0.3689
	14	G*01:01:17	3	0.0049	1	0.0032	2	0.0066	2.0467	0.1846 - 22.6903	0.6207
	15	G*01:01:01:06	2	0.0033	2	0.0065	0	0.0000	0.2026	0.0097 - 4.2385	0.4992
	16	G*01:01:15[compatible]	2	0.0033	0	0.0000	2	0.0066	5.1331	0.2454 - 107.3643	0.2447
	17	G*01:01:09[(no deletion at +615)]	2	0.0033	2	0.0065	0	0.0000	0.2026	0.0097 - 4.2385	0.4992
	18	G*01:01:14	2	0.0033	2	0.0065	0	0.0000	0.2026	0.0097 - 4.2385	0.4992
	19	G*01:01:01:13	2	0.0033	0	0.0000	2	0.0066	5.1331	0.2454 - 107.3643	0.2447
	20	G*01:04:05[compatible]	1	0.0016	1	0.0032	0	0.0000	0.3388	0.0137 - 8.3505	1.0000
	21	G*01:01:30:02	1	0.0016	1	0.0032	0	0.0000	0.3388	0.0137 - 8.3505	1.0000
	22	G*01:01:02:01[(+2798G)]	1	0.0016	0	0.0000	1	0.0033	3.0697	0.1246 - 75.6491	0.4951
	23	G*01:01:02:02	1	0.0016	1	0.0032	0	0.0000	0.3388	0.0137 - 8.3505	1.0000
		**Total [2*n*]**	**610**	**1.0000**	**308**	**1.0000**	**302**	**1.0000**	-	-	-
3′UTR	1	UTR-02	176	0.2885	83	0.2695	93	0.3079	1.2063	0.8494 - 1.7131	0.3257
	**2**	**UTR-01**	**140**	**0.2295**	**60**	**0.1948**	**80**	**0.2649**	**1.4895**	**1.0180 - 2.1793**	**0.0433**
	**3**	**UTR-03**	**81**	**0.1328**	**53**	**0.1721**	**28**	**0.0927**	**0.4917**	**0.3016 - 0.8015**	**0.0041**
	4	UTR-04	75	0.1230	41	0.1331	34	0.1126	0.8262	0.5086 - 1.3421	0.4615
	5	UTR-05	53	0.0869	26	0.0844	27	0.0894	1.0649	0.6061 - 1.8710	0.8861
	6	UTR-06	26	0.0426	10	0.0325	16	0.0530	1.6671	0.7442 - 3.7347	0.2337
	7	UTR-07	26	0.0426	14	0.0455	12	0.0397	0.8690	0.3952 - 1.9107	0.8418
	8	UTR-18	19	0.0311	9	0.0292	10	0.0331	1.1377	0.4557 - 2.8403	0.8195
	9	UTR-08	4	0.0066	4	0.0130	0	0.0000	0.1118	0.0060 - 2.0864	0.1238
	10	UTR-20	3	0.0049	2	0.0065	1	0.0033	0.5083	0.0458 - 5.6353	1.0000
	11	UTR-09	3	0.0049	3	0.0097	0	0.0000	0.1443	0.0074 - 2.8050	0.2488
	12	UTR-17	2	0.0033	2	0.0065	0	0.0000	0.2026	0.0097 - 4.2385	0.4992
	13	UTR-13	1	0.0016	0	0.0000	1	0.0033	3.0697	0.1246 - 75.6491	0.4951
	14	UTR-44	1	0.0016	1	0.0032	0	0.0000	0.3388	0.0137 - 8.3505	1.0000
		**Total [2*n*]**	**610**	**1.0000**	**308**	**1.0000**	**302**	**1.0000**	-	-	-

5′URR: 5′ upstream region. 3′UTR: 3′ untranslated region. OR: Odds Ratio. CI: Confidence Interval. Freq: Frequency. Haplotypes in order of total frequency. Comparisons between PTC patients and controls were made using Fisher's exact test and *P*-values below 0.05 are highlighted in bold.

Table S2. Association between *HLA-G* 5′URR, coding and 3'UTR haplotypes and histopathological features of PTC (n = 185).

		HLA-G haplotypes	Tumor size		Histological Subtype ϕ		Tumor invasion		Extrathyroidal extension		Multicentricity		Metastasis at diagnosis		TNM Staging 8th θ		Hashimoto's thyroiditis		Response to therapy #	
			<2 cm n = 106	≥2 cm n = 79	Low risk n = 152	High risk n = 33	Absent n = 133	Present n = 52	Absent n = 134	Present n = 51	Absent n = 119	Present n = 66	Absent n = 133	Present n = 52	I n = 153	II/III/IV n = 30	Absent n = 132	Present n = 53	Complete n = 111	Incomplete n = 29
			n (freq.)	n (freq.)	n (freq.)	n (freq.)	n (freq.)	n (freq.)	n (freq.)	n (freq.)	n (freq.)	n (freq.)	n (freq.)	n (freq.)	n (freq.)	n (freq.)	n (freq.)	n (freq.)	n (freq.)	n (freq.)
5′URR	1	G010102a	74 (0.3491)	48 (0.3038)	97 (0.3190)	25 (0.3787)	87 (0.3271)	35 (0.3365)	92 (0.3433)	30 (0.2941)	71 (0.2983)	51 (0.3864)	82 (0.3083)	40 (0.3846)	99 (0.3235)	21 (0.3500)	86 (0.3257)	36 (0.3396)	74 (0.3333)	23 (0.3965)
	2	G010101a	50 (0.2358)	33 (0.2089)	66 (0.2171)	17 (0.2575)	58 (0.2180)	25 (0.2404)	61 (0.2276)	22 (0.2157)	52 (0.2185)	31 (0.2348)	61 (0.2293)	22 (0.2115)	72 (0.2352)	11 (0.1833)	56 (0.2121)	27 (0.2547)	52 (0.2342)	9 (0.1551)
	**3**	**G0104a**	18 (0.0849)	23 (0.1456)	35 (0.1151)	6 (0.0909)	31 (0.1165)	10 (0.0962)	27 (0.1007)	14 (0.1373)	**34 [A] (0.1429)**	**7 [A] (0.0530)**	32 (0.1203)	9 (0.0865)	30 (0.0980)	9 (0.1500)	32 (0.1212)	9 (0.0849)	19 (0.0855)	7 (0.1206)
	4	G010101f	15 (0.0708)	12 (0.0759)	23 (0.0756)	4 (0.0606)	17 (0.0639)	10 (0.0962)	19 (0.0709)	8 (0.0784)	17 (0.0714)	10 (0.0758)	19 (0.0714)	8 (0.0769)	21 (0.0686)	6 (0.1000)	20 (0.0757)	7 (0.0660)	18 (0.0810)	4 (0.0689)
	5	G010101b	12 (0.0566)	10 (0.0633)	17 (0.0559)	5 (0.0757)	16 (0.0602)	6 (0.0577)	17 (0.0634)	5 (0.0490)	12 (0.0504)	10 (0.0758)	19 (0.0714)	3 (0.0288)	21 (0.0686)	1 (0.0166)	18 (0.0681)	4 (0.0377)	16 (0.0720)	3 (0.0517)
	6	G0103a	14 (0.0660)	4 (0.0253)	15 (0.0493)	3 (0.0454)	13 (0.0489)	5 (0.0481)	13 (0.0485)	5 (0.0490)	13 (0.0546)	5 (0.0379)	14 (0.0526)	4 (0.0385)	13 (0.0424)	5 (0.0833)	9 (0.0340)	9 (0.0849)	10 (0.0450)	3 (0.0517)
	7	G010101c	8 (0.0377)	9 (0.0570)	13 (0.0427)	4 (0.0606)	15 (0.0564)	2 (0.0192)	15 (0.0560)	2 (0.0196)	13 (0.0546)	4 (0.0303)	11 (0.0414)	6 (0.0577)	15 (0.0490)	2 (0.0333)	10 (0.0378)	7 (0.0660)	13 (0.0585)	2 (0.0344)
	8	G010101d	8 (0.0377)	5 (0.0316)	12 (0.0394)	1 (0.0151)	8 (0.0301)	5 (0.0481)	8 (0.0299)	5 (0.0490)	9 (0.0378)	4 (0.0303)	8 (0.0301)	5 (0.0481)	10 (0.0326)	3 (0.0500)	12 (0.0454)	1 (0.0094)	8 (0.0360)	2 (0.0344)
	9	G0103e	6 (0.0283)	4 (0.0253)	10 (0.0328)	0 (0.0000)	7 (0.0263)	3 (0.0288)	5 (0.0187)	5 (0.0490)	9 (0.0378)	1 (0.0076)	7 (0.0263)	3 (0.0288)	8 (0.0261)	2 (0.0333)	9 (0.0340)	1 (0.0094)	6 (0.0270)	0 (0.0000)
	10	G0103d	3 (0.0142)	4 (0.0253)	6 (0.0197)	1 (0.0151)	7 (0.0263)	0 (0.0000)	6 (0.0224)	1 (0.0098)	3 (0.0126)	4 (0.0303)	6 (0.0226)	1 (0.0096)	7 (0.0228)	0 (0.0000)	7 (0.0265)	0 (0.0000)	4 (0.0180)	3 (0.0517)
	11	G0104b	3 (0.0142)	2 (0.0127)	5 (0.0164)	0 (0.0000)	3 (0.0113)	2 (0.0192)	3 (0.0112)	2 (0.0196)	3 (0.0126)	2 (0.0152)	3 (0.0113)	2 (0.0192)	5 (0.0163)	0 (0.0000)	2 (0.0075)	3 (0.0283)	0 (0.0000)	1 (0.0172)
	12	G010102d	0 (0.0000)	2 (0.0127)	2 (0.0065)	0 (0.0000)	1 (0.0038)	1 (0.0096)	1 (0.0037)	1 (0.0098)	1 (0.0042)	1 (0.0076)	1 (0.0038)	1 (0.0096)	2 (0.0065)	0 (0.0000)	1 (0.0037)	1 (0.0094)	0 (0.0000)	1 (0.0172)
	13	G010101g	1 (0.0047)	0 (0.0000)	1 (0.0032)	0 (0.0000)	1 (0.0038)	0 (0.0000)	1 (0.0037)	0 (0.0000)	0 (0.0000)	1 (0.0076)	1 (0.0038)	0 (0.0000)	1 (0.0032)	0 (0.0000)	0 (0.0000)	1 (0.0094)	1 (0.0045)	0 (0.0000)
	14	G010101j	0 (0.0000)	1 (0.0063)	1 (0.0032)	0 (0.0000)	1 (0.0038)	0 (0.0000)	0 (0.0000)	1 (0.0098)	1 (0.0042)	0 (0.0000)	1 (0.0038)	0 (0.0000)	1 (0.0032)	0 (0.0000)	1 (0.0037)	0 (0.0000)	1 (0.0045)	0 (0.0000)
	15	G010101h	0 (0.0000)	1 (0.0063)	1 (0.0032)	0 (0.0000)	1 (0.0038)	0 (0.0000)	0 (0.0000)	1 (0.0098)	0 (0.0000)	1 (0.0076)	1 (0.0038)	0 (0.0000)	1 (0.0032)	0 (0.0000)	1 (0.0037)	0 (0.0000)	0 (0.0000)	0 (0.0000)
		**Total [2n]**	**212 (1.0000)**	**158 (1.0000)**	**304 (1.0000)**	**66 (1.0000)**	**266 (1.0000)**	**104 (1.0000)**	**268 (1.0000)**	**102 (1.0000)**	**238 (1.0000)**	**132 (1.0000)**	**266 (1.0000)**	**104 (1.0000)**	**306 (1.0000)**	**60 (1.0000)**	**264 (1.0000)**	**106 (1.0000)**	**222 (1.0000)**	**58 (1.0000)**
Coding	1	*G**01:01:01:01	50 (0.2358)	33 (0.2089)	66 (0.2163)	17 (0.2575)	58 (0.2180)	25 (0.2404)	61 (0.2276)	22 (0.2157)	53 (0.2227)	30 (0.2273)	62 (0.2331)	21 (0.2019)	72 (0.2352)	11 (0.1833)	55 (0.2083)	28 (0.2641)	53 (0.2387)	9 (0.1551)
	**2**	***G**01:01:02:01**	39 (0.1840)	31 (0.1962)	57 (0.1868)	13 (0.1969)	50 (0.1880)	20 (0.1923)	52 (0.1940)	18 (0.1765)	41 (0.1723)	29 (0.2197)	48 (0.1805)	22 (0.2115)	56 (0.1830)	13 (0.2166)	54 (0.2045)	16 (0.1509)	**37 [B] (0.1666)**	**17 [B] (0.2931)**
	3	*G**01:01:01:05	19 (0.0896)	18 (0.1139)	29 (0.0950)	8 (0.1212)	29 (0.1090)	8 (0.0769)	31 (0.1157)	6 (0.0588)	24 (0.1008)	13 (0.0985)	28 (0.1053)	9 (0.0865)	34 (0.1111)	3 (0.0500)	26 (0.0984)	11 (0.1037)	27 (0.1216)	5 (0.0862)
	4	*G**01:03:01:02	23 (0.1085)	12 (0.0759)	31 (0.1016)	4 (0.0606)	27 (0.1015)	8 (0.0769)	24 (0.0896)	11 (0.1078)	25 (0.1050)	10 (0.0758)	27 (0.1015)	8 (0.0769)	28 (0.0915)	7 (0.1166)	25 (0.0946)	10 (0.0943)	20 (0.0900)	6 (0.1034)
	**5**	***G**01:04:01**	13 (0.0613)	14 (0.0886)	23 (0.0754)	4 (0.0606)	19 (0.0714)	8 (0.0769)	18 (0.0672)	9 (0.0882)	22 (0.0924)	5 (0.0379)	22 (0.0827)	5 (0.0481)	22 (0.0718)	5 (0.0833)	18 (0.0681)	9 (0.0849)	**9 [C] (0.0405)**	**7 [C] (0.1206)**
	6	*G**01:01:01:04	15 (0.0708)	11 (0.0696)	24 (0.0786)	2 (0.0303)	16 (0.0602)	10 (0.0962)	17 (0.0634)	9 (0.0882)	15 (0.0630)	11 (0.0833)	18 (0.0677)	8 (0.0769)	21 (0.0686)	5 (0.0833)	21 (0.0795)	5 (0.0471)	17 (0.0765)	4 (0.0689)
	7	*G**01:04:04	8 (0.0377)	11 (0.0696)	17 (0.0557)	2 (0.0303)	15 (0.0564)	4 (0.0385)	12 (0.0448)	7 (0.0686)	15 (0.0630)	4 (0.0833)	13 (0.0489)	6 (0.0577)	13 (0.0424)	4 (0.0666)	16 (0.0606)	3 (0.0283)	10 (0.0450)	1 (0.0172)
	8	*G**01:05N	10 (0.0472)	6 (0.0380)	14 (0.0459)	2 (0.0303)	12 (0.0451)	4 (0.0385)	12 (0.0448)	4 (0.0392)	10 (0.0420)	6 (0.0455)	12 (0.0451)	4 (0.0385)	15 (0.0490)	1 (0.0166)	11 (0.0416)	5 (0.0471)	11 (0.0495)	2 (0.0344)
	9	*G**01:06	11 (0.0519)	4 (0.0253)	11 (0.0393)	4 (0.0606)	12 (0.0451)	3 (0.0288)	11 (0.0410)	4 (0.0392)	8 (0.0336)	7 (0.0530)	11 (0.0414)	4 (0.0385)	14 (0.0457)	1 (0.0166)	9 (0.0340)	6 (0.0566)	10 (0.0450)	1 (0.0172)

	10	G*01:01:03:03	8 (0.0377)	6 (0.0380)	11 (0.0393)	3 (0.0454)	10 (0.0376)	4 (0.0385)	11 (0.0410)	3 (0.0294)	10 (0.0420)	4 (0.0833)	8 (0.0301)	6 (0.0577)	11 (0.0359)	3 (0.0500)	12 (0.0454)	2 (0.0188)	10 (0.0450)	2 (0.0344)
	11	G*01:01:01:08	6 (0.0283)	3 (0.0190)	9 (0.0295)	0 (0.0000)	5 (0.0188)	4 (0.0385)	6 (0.0224)	3 (0.0294)	6 (0.0252)	3 (0.0227)	6 (0.0226)	3 (0.0288)	8 (0.0261)	1 (0.0166)	9 (0.0340)	0 (0.0000)	6 (0.0270)	1 (0.0172)
	12	G*01:01:22:01	6 (0.0283)	1 (0.0063)	6 (0.0196)	1 (0.0151)	5 (0.0188)	2 (0.0192)	5 (0.0187)	2 (0.0196)	1 (0.0042)	6 (0.0455)	4 (0.0150)	3 (0.0288)	6 (0.0196)	0 (0.0000)	2 (0.0075)	5 (0.0471)	3 (0.0135)	2 (0.0344)
	13	G*01:01:01:15	2 (0.0094)	2 (0.0127)	3 (0.0098)	1 (0.0151)	3 (0.0113)	1 (0.0096)	2 (0.0075)	2 (0.0196)	3 (0.0126)	1 (0.0076)	2 (0.0075)	2 (0.0192)	2 (0.0065)	2 (0.0333)	3 (0.0113)	1 (0.0094)	2 (0.0090)	1 (0.0172)
	14	G*01:01:15 compatible	1 (0.0047)	1 (0.0063)	1 (0.0032)	1 (0.0151)	2 (0.0075)	0 (0.0000)	1 (0.0037)	1 (0.0098)	1 (0.0042)	1 (0.0076)	2 (0.0075)	0 (0.0000)	2 (0.0065)	0 (0.0000)	2 (0.0075)	0 (0.0000)	2 (0.0090)	0 (0.0000)
	15	G*01:01:01:13	1 (0.0047)	1 (0.0063)	0 (0.0000)	2 (0.0303)	2 (0.0075)	0 (0.0000)	2 (0.0075)	0 (0.0000)	2 (0.0084)	0 (0.0000)	2 (0.0075)	0 (0.0000)	1 (0.0032)	1 (0.0166)	0 (0.0000)	2 (0.0188)	2 (0.0090)	0 (0.0000)
	16	G*01:01:17	0 (0.0000)	2 (0.0127)	1 (0.0032)	1 (0.0151)	0 (0.0000)	2 (0.0192)	2 (0.0075)	0 (0.0000)	1 (0.0042)	1 (0.0076)	0 (0.0000)	2 (0.0192)	0 (0.0000)	2 (0.0333)	0 (0.0000)	2 (0.0188)	2 (0.0090)	0 (0.0000)
	17	G*01:01:01:06	0 (0.0000)	1 (0.0063)	1 (0.0032)	0 (0.0000)	1 (0.0038)	0 (0.0000)	0 (0.0000)	1 (0.0098)	0 (0.0000)	1 (0.0076)	1 (0.0038)	0 (0.0000)	1 (0.0032)	0 (0.0000)	1 (0.0037)	0 (0.0000)	0 (0.0000)	0 (0.0000)
	18	G*01:01:02:01(+2798G)	0 (0.0000)	1 (0.0063)	0 (0.0000)	1 (0.0151)	0 (0.0000)	1 (0.0096)	1 (0.0037)	0 (0.0000)	1 (0.0042)	0 (0.0000)	0 (0.0000)	1 (0.0096)	0 (0.0000)	1 (0.0166)	0 (0.0000)	1 (0.0094)	1 (0.0045)	0 (0.0000)
		**Total [2*n*]**	**212 (1.0000)**	**158 (1.0000)**	**304 (1.0000)**	**66 (1.0000)**	**266 (1.0000)**	**104 (1.0000)**	**268 (1.0000)**	**102 (1.0000)**	**238 (1.0000)**	**132 (1.0000)**	**266 (1.0000)**	**104 (1.0000)**	**306 (1.0000)**	**60 (1.0000)**	**264 (1.0000)**	**106 (1.0000)**	**222 (1.0000)**	**58 (1.0000)**
**3′UTR**	**1**	**UTR-02**	66 (0.3113)	45 (0.2848)	89 (0.2927)	22 (0.3333)	79 (0.2970)	32 (0.3077)	83 (0.3097)	28 (0.2745)	**62 [D] (0.2605)**	**49 [D] (0.3712)**	75 (0.2820)	36 (0.3462)	91 (0.2973)	18 (0.3000)	76 (0.2878)	35 (0.3301)	64 (0.2882)	22 (0.3793)
	2	UTR-01	56 (0.2642)	38 (0.2405)	77 (0.2532)	17 (0.2575)	66 (0.2481)	28 (0.2692)	67 (0.2500)	27 (0.2647)	63 (0.2647)	31 (0.2348)	69 (0.2594)	25 (0.2404)	80 (0.2614)	14 (0.2333)	65 (0.2462)	29 (0.2735)	60 (0.2702)	11 (0.1896)
	**3**	**UTR-03**	21 (0.0991)	24 (0.1519)	39 (0.1282)	6 (0.0909)	34 (0.1278)	11 (0.1058)	30 (0.1119)	15 (0.1471)	**36 [E] (0.1513)**	**9 [E] (0.0682)**	35 (0.1316)	10 (0.0962)	35 (0.1143)	8 (0.1333)	34 (0.1287)	11 (0.1037)	19 (0.0855)	7 (0.1206)
	4	UTR-04	19 (0.0896)	19 (0.1203)	29 (0.0953)	9 (0.1363)	29 (0.1090)	9 (0.0865)	31 (0.1157)	7 (0.0686)	23 (0.0966)	15 (0.1136)	29 (0.1090)	9 (0.0865)	35 (0.1143)	3 (0.0500)	27 (0.1022)	11 (0.1037)	27 (0.1216)	5 (0.0862)
	5	UTR-05	23 (0.1085)	12 (0.0759)	31 (0.1019)	4 (0.0606)	27 (0.1015)	8 (0.0769)	24 (0.0896)	11 (0.1078)	25 (0.1050)	10 (0.0758)	27 (0.1015)	8 (0.0769)	28 (0.0915)	7 (0.1166)	25 (0.0946)	10 (0.0943)	20 (0.0900)	6 (0.1034)
	6	UTR-06	11 (0.0519)	7 (0.0443)	14 (0.0460)	4 (0.0606)	12 (0.0451)	6 (0.0577)	14 (0.0522)	4 (0.0392)	10 (0.0420)	8 (0.0606)	13 (0.0489)	5 (0.0481)	16 (0.0522)	2 (0.0333)	15 (0.0568)	3 (0.0283)	13 (0.0585)	2 (0.0344)
	7	UTR-07	8 (0.0377)	6 (0.0380)	11 (0.0361)	3 (0.0454)	10 (0.0376)	4 (0.0385)	11 (0.0410)	3 (0.0294)	10 (0.0420)	4 (0.0303)	8 (0.0301)	6 (0.0577)	11 (0.0359)	3 (0.0500)	12 (0.0454)	2 (0.0188)	10 (0.0450)	2 (0.0344)
	8	UTR-18	7 (0.0330)	5 (0.0316)	11 (0.0361)	1 (0.0151)	8 (0.0301)	4 (0.0385)	8 (0.0299)	4 (0.0392)	6 (0.0252)	6 (0.0455)	8 (0.0301)	4 (0.0385)	8 (0.0261)	4 (0.0666)	8 (0.0303)	4 (0.0377)	9 (0.0405)	1 (0.0172)
	9	UTR-20	1 (0.0047)	1 (0.0063)	2 (0.0065)	0 (0.0000)	1 (0.0038)	1 (0.0096)	0 (0.0000)	2 (0.0196)	2 (0.0084)	0 (0.0000)	2 (0.0075)	0 (0.0000)	2 (0.0065)	0 (0.0000)	2 (0.0075)	0 (0.0000)	0 (0.0000)	1 (0.0172)
	10	UTR-13	0 (0.0000)	1 (0.0063)	1 (0.0032)	0 (0.0000)	0 (0.0000)	1 (0.0096)	0 (0.0000)	1 (0.0098)	1 (0.0042)	0 (0.0000)	0 (0.0000)	1 (0.0096)	0 (0.0000)	1 (0.0166)	0 (0.0000)	1 (0.0094)	0 (0.0000)	1 (0.0172)
		**Total [2*n*]**	**212 (1.0000)**	**158 (1.0000)**	**304 (1.0000)**	**66 (1.0000)**	**266 (1.0000)**	**104 (1.0000)**	**268 (1.0000)**	**102 (1.0000)**	**238 (1.0000)**	**132 (1.0000)**	**266 (1.0000)**	**104 (1.0000)**	**306 (1.0000)**	**60 (1.0000)**	**264 (1.0000)**	**106 (1.0000)**	**222 (1.0000)**	**58 (1.0000)**

5′URR: 5′ upstream region. 3′UTR: 3′ untranslated region. Freq: Frequency. Haplotypes in order of total frequency. Association of haplotypes with histopathological features of PTC was made by using Fisher's exact test and P-values below 0.05 are highlighted in bold. ϕ Low-risk/more frequent subtypes (Classic + Follicular) or high-risk/less frequent subtypes (Oncocytic, solid, trabecular and others). θ Information not available for 2 PTC patients (total *n* = 183). Tumor node metastasis (TNM) system refers to the primary tumor, lymph node metastasis and distant metastasis. # Information not available for 45 PTC patients (total *n* = 140). Complete (the sum of patients presenting excellent and indeterminate responses) or incomplete (the sum of patients presenting biochemical and structural incomplete responses).

[A] OR: 0.3360; 95% CI: 0.1446-0.7810; *P*: 0.0089. [B] OR: 2.0732; 95% CI: 1.0646-4.0374; *P*: 0.0392. [C] OR: 3.2484; 95% CI: 1.1552-9.1345; *P*: 0.0279. [D] OR: 1.6759; 95% CI: 1.0616-2.6456; *P*: 0.0328.

[E] OR: 0.4106; 95% CI: 0.1912-0.8815; P: 0.0200.

OR: Odds ratio. CI: Confidence interval.

Table S3. Association between *HLA-G* extended haplotypes and histopathological features of PTC (n = 185) (Continuation of **Table 2**).

**HLA-G extended haplotypes**				**Tumor size**		**Histological Subtype ϕ**		**Tumor invasion**		**Extrathyroidal extension**		**Multicentricity**		**Metastasis at diagnosis**		**TNM Staging 8th θ**		**Hashimoto's thyroiditis**		**Response to therapy #**	
	**5′URR**	**Coding**	**3′UTR**	**<2 cm** n **= 106** n **(freq.)**	**≥2 cm** n **= 79** n **(freq.)**	**Low risk** n **= 152** n **(freq.)**	**High risk** n **= 33** n **(freq.)**	**Absent** n **= 133** n **(freq.)**	**Present** n **= 52** n **(freq.)**	**Absent** n **= 134** n **(freq.)**	**Present** n **= 51** n **(freq.)**	**Absent** n **= 119** n **(freq.)**	**Present** n **= 66** n **(freq.)**	**Absent** n **= 133** n **(freq.)**	**Present** n **= 52** n **(freq.)**	**I** n **= 153** n **(freq.)**	**II/III/IV** n **= 30** n **(freq.)**	**Absent** n **= 132** n **(freq.)**	**Present** n **= 53** n **(freq.)**	**Complete** n **= 111** n **(freq.)**	**Incomplete** n **= 29** n **(freq.)**
18	G010101d	01:01:01:15	UTR-01	2 (0.0094)	2 (0.0127)	3 (0.0098)	1 (0.0151)	3 (0.0113)	1 (0.0096)	2 (0.0075)	2 (0.0196)	3 (0.0126)	1 (0.0076)	2 (0.0075)	2 (0.0192)	2 (0.0065)	2 (0.0333)	3 (0.0113)	1 (0.0094)	2 (0.0090)	1 (0.0172)
19	G010101f	01:01:01:04	UTR-20	1 (0.0047)	1 (0.0063)	2 (0.0065)	0 (0.0000)	1 (0.0038)	1 (0.0096)	0 (0.0000)	2 (0.0196)	2 (0.0084)	0 (0.0000)	2 (0.0075)	0 (0.0000)	2 (0.0065)	0 (0.0000)	2 (0.0075)	0 (0.0000)	0 (0.0000)	1 (0.0172)
20	G010101a	01:01:01:01	UTR-06	2 (0.0094)	0 (0.0000)	2 (0.0065)	0 (0.0000)	1 (0.0038)	1 (0.0096)	2 (0.0075)	0 (0.0000)	0 (0.0000)	2 (0.0152)	1 (0.0038)	1 (0.0096)	2 (0.0065)	0 (0.0000)	2 (0.0075)	0 (0.0000)	1 (0.0045)	0 (0.0000)
21	G010102d	01:01:02:01	UTR-02	0 (0.0000)	2 (0.0127)	2 (0.0065)	0 (0.0000)	1 (0.0038)	1 (0.0096)	1 (0.0037)	1 (0.0098)	1 (0.0042)	1 (0.0076)	1 (0.0038)	1 (0.0096)	2 (0.0065)	0 (0.0000)	1 (0.0037)	1 (0.0094)	0 (0.0000)	1 (0.0172)
22	G010102a	01:01:17	UTR-02	0 (0.0000)	2 (0.0127)	1 (0.0032)	1 (0.0151)	0 (0.0000)	2 (0.0192)	2 (0.0075)	0 (0.0000)	1 (0.0042)	1 (0.0076)	0 (0.0000)	2 (0.0192)	0 (0.0000)	2 (0.0333)	0 (0.0000)	2 (0.0188)	2 (0.0090)	0 (0.0000)
23	G010101b	01:01:15 compatible	UTR-06	1 (0.0047)	1 (0.0063)	1 (0.0032)	1 (0.0151)	2 (0.0075)	0 (0.0000)	1 (0.0037)	1 (0.0098)	1 (0.0042)	1 (0.0076)	2 (0.0075)	0 (0.0000)	2 (0.0065)	0 (0.0000)	2 (0.0075)	0 (0.0000)	2 (0.0090)	0 (0.0000)
24	G010101f	01:01:01:13	UTR-06	1 (0.0047)	1 (0.0063)	0 (0.0000)	2 (0.0303)	2 (0.0075)	0 (0.0000)	2 (0.0075)	0 (0.0000)	2 (0.0084)	0 (0.0000)	2 (0.0075)	0 (0.0000)	1 (0.0032)	1 (0.0166)	0 (0.0000)	2 (0.0188)	2 (0.0090)	0 (0.0000)
25	G0104a	01:04:01	UTR-13	0 (0.0000)	1 (0.0063)	1 (0.0032)	0 (0.0000)	0 (0.0000)	1 (0.0096)	0 (0.0000)	1 (0.0098)	1 (0.0042)	0 (0.0000)	0 (0.0000)	1 (0.0096)	0 (0.0000)	1 (0.0166)	0 (0.0000)	1 (0.0094)	0 (0.0000)	1 (0.0172)
26	G010101g	01:01:01:01	UTR-01	1 (0.0047)	0 (0.0000)	1 (0.0032)	0 (0.0000)	1 (0.0038)	0 (0.0000)	1 (0.0037)	0 (0.0000)	0 (0.0000)	1 (0.0076)	1 (0.0038)	0 (0.0000)	1 (0.0032)	0 (0.0000)	0 (0.0000)	1 (0.0094)	1 (0.0045)	0 (0.0000)
27	G010102a	01:01:02:01 (+2798G)	UTR-02	0 (0.0000)	1 (0.0063)	0 (0.0000)	1 (0.0151)	0 (0.0000)	1 (0.0096)	1 (0.0037)	0 (0.0000)	1 (0.0042)	0 (0.0000)	0 (0.0000)	1 (0.0096)	0 (0.0000)	1 (0.0166)	0 (0.0000)	1 (0.0094)	1 (0.0045)	0 (0.0000)
28	G010101a	01:01:02:01	UTR-02	0 (0.0000)	1 (0.0063)	1 (0.0032)	0 (0.0000)	1 (0.0038)	0 (0.0000)	1 (0.0037)	0 (0.0000)	0 (0.0000)	1 (0.0076)	0 (0.0000)	1 (0.0096)	1 (0.0032)	0 (0.0000)	1 (0.0037)	0 (0.0000)	0 (0.0000)	0 (0.0000)
29	G010101a	01:01:01:04	UTR-18	1 (0.0047)	0 (0.0000)	1 (0.0032)	0 (0.0000)	1 (0.0038)	0 (0.0000)	0 (0.0000)	1 (0.0098)	0 (0.0000)	1 (0.0076)	1 (0.0038)	0 (0.0000)	1 (0.0032)	0 (0.0000)	1 (0.0037)	0 (0.0000)	1 (0.0045)	0 (0.0000)
30	G010101j	01:01:01:01	UTR-01	0 (0.0000)	1 (0.0063)	1 (0.0032)	0 (0.0000)	1 (0.0038)	0 (0.0000)	0 (0.0000)	1 (0.0098)	1 (0.0042)	0 (0.0000)	1 (0.0038)	0 (0.0000)	1 (0.0032)	0 (0.0000)	1 (0.0037)	0 (0.0000)	1 (0.0045)	0 (0.0000)
31	G010101a	01:01:01:01	UTR-04	1 (0.0047)	0 (0.0000)	0 (0.0000)	1 (0.0151)	0 (0.0000)	1 (0.0096)	1 (0.0037)	0 (0.0000)	0 (0.0000)	1 (0.0076)	1 (0.0038)	0 (0.0000)	1 (0.0032)	0 (0.0000)	1 (0.0037)	0 (0.0000)	1 (0.0045)	0 (0.0000)
32	G010101b	01:01:01:05	UTR-01	1 (0.0047)	0 (0.0000)	1 (0.0032)	0 (0.0000)	1 (0.0038)	0 (0.0000)	1 (0.0037)	0 (0.0000)	1 (0.0042)	0 (0.0000)	1 (0.0038)	0 (0.0000)	1 (0.0032)	0 (0.0000)	1 (0.0037)	0 (0.0000)	1 (0.0045)	0 (0.0000)
33	G010101h	01:01:01:06	UTR-04	0 (0.0000)	1 (0.0063)	1 (0.0032)	0 (0.0000)	1 (0.0038)	0 (0.0000)	0 (0.0000)	1 (0.0098)	0 (0.0000)	1 (0.0076)	1 (0.0038)	0 (0.0000)	1 (0.0032)	0 (0.0000)	1 (0.0037)	0 (0.0000)	0 (0.0000)	0 (0.0000)
**Total [2n] ***				**212 (1.0000)**	**158 (1.0000)**	**304 (1.0000)**	**66 (1.0000)**	**266 (1.0000)**	**104 (1.0000)**	**268 (1.0000)**	**102 (1.0000)**	**238 (1.0000)**	**132 (1.0000)**	**266 (1.0000)**	**104 (1.0000)**	**306 (1.0000)**	**60 (1.0000)**	**264 (1.0000)**	**106 (1.0000)**	**222 (1.0000)**	**58 (1.0000)**

5′URR: 5′ upstream region. 3′UTR: 3′ untranslated region. Freq: Frequency. Haplotypes in order of total frequency. Association of haplotypes with histopathological features of PTC was made by using Fisher's exact test and *P*-values below 0.05 are highlighted in bold. ϕ Low-risk/more frequent subtypes (Classic + Follicular) or high-risk/less frequent subtypes (Oncocytic, solid, trabecular and others). θ Information not available for 2 PTC patients (total n = 183). Tumor node metastasis (TNM) system refers to the primary tumor, lymph node metastasis and distant metastasis. # Information not available for 45 PTC patients (total n = 140). Complete (the sum of patients presenting excellent and indeterminate responses) or incomplete (the sum of patients presenting biochemical and structural incomplete responses). * Total [2n] refers to the sum of all haplotypes within both Table 2 and Table S3.

Table S4. *HLA-G* 5′URR, coding and 3'UTR haplotypes in papillary thyroid carcinoma (PTC) patients according to the expression of HLA-G in the tumor microenvironment [n = 170].

				HLA-G tumor expression #						
	***HLA-G* haplotypes**			**Low**		**High**		**OR**	**95% CI**	***P***
			n	***n***	**Freq.**	***n***	**Freq.**			
**5′URR**	1	G010102a	115	37	0.3627	78	0.3277	0.8564	0.5267 - 1.3923	0.5344
	2	G010101a	75	21	0.2059	54	0.2269	1.1320	0.6415 - 1.9972	0.7755
	3	G0104a	37	15	0.1471	22	0.0924	0.5907	0.2927 - 1.1918	0.1822
	4	G010101f	27	7	0.0686	20	0.0840	1.2451	0.5093 - 3.0435	0.8270
	5	G010101b	19	6	0.0588	13	0.0546	0.9244	0.3413 - 2.5038	1.0000
	6	G0103a	16	5	0.0490	11	0.0462	0.9401	0.3181 - 2.7780	1.0000
	**7**	**G010101c**	15	**1**	**0.0098**	**14**	**0.0588**	**6.3125**	**0.8189 - 48.6592**	**0.0457**
	8	G010101d	12	3	0.0294	9	0.0378	1.2969	0.3437 - 4.8927	1.0000
	9	G0103e	8	2	0.0196	6	0.0252	1.2931	0.2565 - 6.5171	1.0000
	10	G0103d	6	1	0.0098	5	0.0210	2.1674	0.2500 - 18.7878	0.6729
	11	G0104b	5	2	0.0196	3	0.0126	0.6383	0.1050 - 3.8786	0.6383
	12	G010102d	2	1	0.0098	1	0.0042	0.4262	0.0263 - 6.8801	0.5106
	13	G010101g	1	1	0.0098	0	0.0000	0.1419	0.0057 - 3.5117	0.3000
	14	G010101j	1	0	0.0000	1	0.0042	1.2947	0.0523 - 32.0496	1.0000
	15	G010101h	1	0	0.0000	1	0.0042	1.2947	0.0523 - 32.0496	1.0000
		**Total [2*n*]**	**340**	**102**	**1.0000**	**238**	**1.0000**	**-**	**-**	**-**
**Coding**	1	*G**01:01:01:01	76	21	0.2059	55	0.2311	1.1593	0.6577 - 2.0431	0.6713
	**2**	***G**01:01:02:01**	67	**29**	**0.2843**	**38**	**0.1597**	**0.4783**	**0.2752 - 0.8311**	**0.0111**
	3	*G**01:01:01:05	32	7	0.0686	25	0.1050	1.5929	0.6658 - 3.8108	0.4173
	4	*G**01:03:01:02	30	8	0.0784	22	0.0924	1.1968	0.5142 - 2.7850	0.8353
	5	*G**01:01:01:04	25	6	0.0588	19	0.0798	1.3881	0.5375 - 3.5845	0.6513
	6	*G**1:04:01	23	11	0.1078	12	0.0504	0.4393	0.1870 - 1.0313	0.0616
	7	*G**1:04:04	19	6	0.0588	13	0.0546	0.9244	0.3413 - 2.5038	1.0000
	8	*G**1:06	15	4	0.0392	11	0.0462	1.1872	0.3689 - 3.8200	1.0000
	9	*G**01:05N	13	2	0.0196	11	0.0462	2.4229	0.5273 - 11.1323	0.3583
	10	*G**01:01:03:03	13	2	0.0196	11	0.0462	2.4229	0.5273 - 11.1323	0.3583
	11	*G**01:01:01:08	8	3	0.0294	5	0.0210	0.7082	0.1660 - 3.0205	0.7007
	12	*G**01:01:22:01	7	0	0.0000	7	0.0294	6.6415	0.3757 - 117.3865	0.1076
	13	*G**01:01:01:15	4	0	0.0000	4	0.0168	3.9339	0.2098 - 73.7422	0.3207
	14	*G**01:01:15^{compatible}	2	0	0.0000	2	0.0084	2.1670	0.1031 - 45.5380	1.0000
	15	*G**01:01:01:13	2	1	0.0098	1	0.0042	0.4262	0.0263 - 6.8801	0.5106
	16	*G**01:01:17	2	1	0.0098	1	0.0042	0.4262	0.0263 - 6.8801	0.5106
	17	*G**01:01:01:06	1	0	0.0000	1	0.0042	1.2947	0.0523 - 32.0496	1.0000
	18	*G**01:01:02:01$^{(+2798G)}$	1	1	0.0098	0	0.0000	0.1419	0.0057 - 3.5117	0.3000
		**Total [2*n*]**	**340**	**102**	**1.0000**	**238**	**1.0000**	**-**	**-**	**-**
**3′UTR**	1	UTR-02	105	37	0.3627	68	0.2857	0.7027	0.4296 - 1.1492	0.1616
	2	UTR-01	86	23	0.2255	63	0.2647	1.2365	0.7159 - 2.1355	0.4975
	3	UTR-03	41	16	0.1569	25	0.1050	0.6309	0.3210 - 1.2398	0.2039
	4	UTR-04	33	7	0.0686	26	0.1092	1.6644	0.6980 - 3.9686	0.3186
	5	UTR-05	30	8	0.0784	22	0.0924	1.1968	0.5142 - 2.7850	0.8353
	6	UTR-06	18	5	0.0490	13	0.0546	1.1209	0.3889 - 3.2305	1.0000
	7	UTR-07	13	2	0.0196	11	0.0462	2.4229	0.5273 - 11.1323	0.3583
	8	UTR-18	11	3	0.0294	8	0.0336	1.1478	0.2982 - 4.4172	1.0000
	9	UTR-20	2	0	0.0000	2	0.0084	2.1670	0.1031 - 45.5380	1.0000
	10	UTR-13	1	1	0.0098	0	0.0000	0.1419	0.0057 - 3.5117	0.3000
		**Total [2*n*]**	**340**	**102**	**1.0000**	**238**	**1.0000**	**-**	**-**	**-**

5′URR: 5′ upstream region. 3′UTR: 3′ untranslated region. OR: Odds Ratio. CI: Confidence Interval. Freq: Frequency. Haplotypes in order of total frequency. Association of haplotypes with HLA-G tumor expression was made by using Fisher's exact test and *P*-values below 0.05 are highlighted in bold. # Low (≤ 50% of positive tumor cells) or high (> 50% of positive tumor cells).

Table S5. *HLA-G* 5′URR, coding and 3'UTR haplotypes in papillary thyroid carcinoma (PTC) patients according to sHLA-G detection in plasma before thyroidectomy [n = 84].

		***HLA-G* haplotypes**		**sHLA-G**				**OR**	**95% CI**	***P***
				**Undetectable**		**Detectable**				
			n	***n***	**Freq.**	***n***	**Freq.**			
**5′URR**	1	G010102a	59	33	0.4125	26	0.2955	0.5973	0.3153 - 1.1311	0.1452
	2	G010101a	30	12	0.1500	18	0.2045	1.4571	0.6527 - 3.2528	0.4221
	3	G0104a	18	8	0.1000	10	0.1136	1.1538	0.4316 - 3.0846	0.8081
	4	G010101f	14	6	0.0750	8	0.0909	1.2333	0.4086 - 3.7225	0.7850
	5	G010101b	8	4	0.0500	4	0.0455	0.9048	0.2186 - 3.7439	1.0000
	6	G0103a	8	2	0.0250	6	0.0682	2.8537	0.5590 - 14.5654	0.2817
	7	G010101c	7	3	0.0375	4	0.0455	1.2222	0.2650 - 5.6362	1.0000
	8	G010101d	7	3	0.0375	4	0.0455	1.2222	0.2650 - 5.6362	1.0000
	9	G0103e	7	3	0.0375	4	0.0455	1.2222	0.2650 - 5.6362	1.0000
	10	G0104b	5	3	0.0375	2	0.0227	0.5969	0.0971 - 3.6671	0.6698
	11	G0103d	4	3	0.0375	1	0.0114	0.2950	0.0300 - 2.8954	0.3480
	12	G010101h	1	0	0.0000	1	0.0114	2.7600	0.1108 - 68.7254	1.0000
		**Total [2*n*]**	**168**	**80**	**1.0000**	**88**	**1.0000**	**-**	**-**	**-**
**Coding**	1	*G**01:01:01:01	29	12	0.1500	17	0.1932	1.3568	0.6033 - 3.0510	0.5417
	**2**	***G**01:01:02:01**	**28**	**19**	**0.2375**	**9**	**0.1023**	**0.3658**	**0.1546 - 0.8648**	**0.0228**
	3	*G**01:03:01:02	19	8	0.1000	11	0.1250	1.2857	0.4894 - 3.3772	0.6355
	4	*G**01:01:01:05	15	7	0.0875	8	0.0909	1.0429	0.3602 - 3.0185	1.0000
	5	*G**1:04:01	15	6	0.0750	9	0.1023	1.4051	0.4768 - 4.1397	0.5968
	6	*G**01:01:01:04	15	6	0.0750	9	0.1023	1.4051	0.4768 - 4.1397	0.5968
	7	*G**1:04:04	8	5	0.0625	3	0.0341	0.5294	0.1223 - 2.2902	0.4802
	8	*G**01:05N	8	4	0.0500	4	0.0455	0.9048	0.2186 - 3.7439	1.0000
	9	*G**1:06	8	6	0.0750	2	0.0227	0.2868	0.0561 - 1.4641	0.1528
	10	*G**01:01:03:03	7	2	0.0250	5	0.0568	2.3494	0.4428 - 12.4641	0.4470
	11	*G**01:01:01:08	5	3	0.0375	2	0.0227	0.5969	0.0971 - 3.6671	0.6698
	12	*G**01:01:22:01	5	2	0.0250	3	0.0341	1.3765	0.2240 - 8.4563	1.0000
	13	*G**01:01:01:15	2	0	0.0000	2	0.0227	4.6532	0.2200 - 98.4006	0.4981
	14	*G**01:01:17	2	0	0.0000	2	0.0227	4.6532	0.2200 - 98.4006	0.4981
	15	*G**01:01:01:06	1	0	0.0000	1	0.0114	2.7600	0.1108 - 68.7254	1.0000
	16	*G**01:01:02:01$^{(+2798G)}$	1	0	0.0000	1	0.0114	2.7600	0.1108 - 68.7254	1.0000
		**Total [2*n*]**	**168**	**80**	**1.0000**	**88**	**1.0000**	**-**	**-**	**-**
**3′UTR**	**1**	**UTR-02**	**52**	**31**	**0.3875**	**21**	**0.2386**	**0.4954**	**0.2547 - 0.9636**	**0.0453**
	2	UTR-01	35	14	0.1750	21	0.2386	1.4776	0.6931 - 3.1496	0.3458
	3	UTR-03	23	11	0.1375	12	0.1364	0.9904	0.4105 - 2.3894	1.0000
	4	UTR-05	19	8	0.1000	11	0.1250	1.2857	0.4894 - 3.3772	0.6355
	5	UTR-04	16	8	0.1000	8	0.0909	0.9000	0.3211 - 2.5219	1.0000
	6	UTR-06	9	5	0.0625	4	0.0455	0.7143	0.1849 - 2.7584	0.7377
	7	UTR-07	7	2	0.0250	5	0.0568	2.3494	0.4428 - 12.4641	0.4470
	**8**	**UTR-18**	**6**	**0**	**0.0000**	**6**	**0.0682**	**12.6848**	**0.7030 - 228.8785**	**0.0295**
	9	UTR-20	1	1	0.0125	0	0.0000	0.2994	0.0120 - 7.4562	0.4762
		**Total [2*n*]**	**168**	**80**	**1.0000**	**88**	**1.0000**	**-**	**-**	**-**

5′URR: 5′ upstream region. 3′UTR: 3′ untranslated region. OR: Odds Ratio. CI: Confidence Interval. Freq: Frequency. Haplotypes in order of total frequency. Association of haplotypes with sHLA-G detection was made by using Fisher's exact test and *P*-values below 0.05 are highlighted in bold.
